# A cell cycle-independent mode of the Rad9-Dpb11 interaction is induced by DNA damage

**DOI:** 10.1038/s41598-017-11937-z

**Published:** 2017-09-14

**Authors:** Giulia di Cicco, Susanne C. S. Bantele, Karl-Uwe Reusswig, Boris Pfander

**Affiliations:** Max Planck Institute of Biochemistry, DNA Replication and Genome Integrity, Martinsried, Germany

## Abstract

Budding yeast Rad9, like its orthologs, controls two aspects of the cellular response to DNA double strand breaks (DSBs) – signalling of the DNA damage checkpoint and DNA end resection. Rad9 binds to damaged chromatin via modified nucleosomes independently of the cell cycle phase. Additionally, Rad9 engages in a cell cycle-regulated interaction with Dpb11 and the 9-1-1 clamp, generating a second pathway that recruits Rad9 to DNA damage sites. Binding to Dpb11 depends on specific S/TP phosphorylation sites of Rad9, which are modified by cyclin-dependent kinase (CDK). Here, we show that these sites additionally become phosphorylated upon DNA damage. We define the requirements for DNA damage-induced S/TP phosphorylation of Rad9 and show that it is independent of the cell cycle or CDK activity but requires prior recruitment of Rad9 to damaged chromatin, indicating that it is catalysed by a chromatin-bound kinase. The checkpoint kinases Mec1 and Tel1 are required for Rad9 S/TP phosphorylation, but their influence is likely indirect and involves phosphorylation of Rad9 at S/TQ sites. Notably, DNA damage-induced S/TP phosphorylation triggers Dpb11 binding to Rad9, but the DNA damage-induced Rad9-Dpb11 interaction is dispensable for recruitment to DNA damage sites, indicating that the Rad9-Dpb11 interaction functions beyond Rad9 recruitment.

## Introduction

DNA damage (such as double strand breaks (DSBs)) elicits cellular signalling pathways, collectively known as the DNA damage response (reviewed in ref. 1). Among these, checkpoint mechanisms control cell cycle progression as well as transcriptional and post-translational regulation of DNA repair and replication. Furthermore, local signalling events are critical in directing DNA repair pathway choice. Budding yeast Rad9 was the first checkpoint protein to be discovered^2^. Since then, it has become evident that Rad9, as well as its orthologs such as fission yeast Crb2^3, 4^ and human 53BP1 (reviewed in ref. 5), play a crucial role in the DNA damage response, having at least two functions: signal transduction in the DNA damage checkpoint (reviewed in ref. 1) and control of DNA end resection, a local process that critically determines DSB repair pathway choice (reviewed in ref. 6).

As checkpoint signalling mediator, Rad9 links the signal transduction from the apical kinase Mec1 to the effector kinase Rad53^7–12^. As such, it is essential for activation of Rad53 and therefore for the activation of a global checkpoint response upon DNA damage. Moreover, Rad9 is also an inhibitor of DNA end resection^13–16^. Since DNA end resection generates the DNA substrate for recombination-based repair and interferes with ligation-based repair, Rad9 is a critical regulator of DSB repair pathway choice. To fulfil these two functions, Rad9 engages in several protein-protein interactions that occur within damaged chromatin^17–22^.

Rad9 binds to modified histones via two distinct domains. The TUDOR domain of Rad9 interacts with histone H3 in its K79-methylated form^19, 22^, a widespread modification of chromatin that is introduced by the methyltransferase Dot1^23, 24^. The tandem-BRCT domain of Rad9 interacts with histone H2A in its S129-phosphorylated form (γH2A^21, 25^), a DNA damage-specific chromatin mark introduced by the apical checkpoint kinases Mec1 and Tel1^26^. As such, Rad9 is a bivalent nucleosome binder, a feature that is conserved among Rad9 orthologs, even though different histone marks are being recognized^27–31^.

Rad9 also binds to the scaffold protein Dpb11^17, 18^. Dpb11 contains two pairs of BRCT domains, which provide two phospho-protein binding surfaces (reviewed in ref. 32). While Rad9 binds to BRCT1 + 2, Dpb11 also interacts with the 9-1-1 complex via BRCT3 + 4^17, 33, 34^. Physical and genetic interaction data suggest that these interactions generate a second pathway that recruits Rad9 to DNA damage sites: DNA damage-loaded 9-1-1 can tether Dpb11, which in turn can recruit Rad9^17, 33^. Notably, the interaction of Dpb11 with Rad9 depends on Rad9 phosphorylation at S462 and T474 residues^17^. Both sites match the minimal consensus (S/TP) for phosphorylation by cyclin-dependent kinase (Cdc28, in the following referred to as CDK) and consistently a CDK-dependent interaction between Rad9 and Dpb11 can be observed in G2/M-arrested cells^17^.

Furthermore, Rad9 binds to the checkpoint effector kinase Rad53^7, 8, 10, 12^. This interaction involves phosphorylation of Rad9 in the S/TQ cluster domain (SCD), which is specifically bound by the FHA domains of Rad53. Rad9 is phosphorylated in the SCD by the apical kinases Mec1 and Tel1 upon association with damaged chromatin^7, 12^. Current models suggest that Rad53 is transiently recruited to damaged chromatin by this mechanism (reviewed in ref. 1). Here, it becomes activated by Mec1/Tel1 phosphorylation, before it dissociates from the DNA damage site to set off the global DNA damage response.

Promoting Rad53 phosphorylation and activation offers a straightforward mechanism of how Rad9 mediates checkpoint signalling. In contrast, it is less clear by which mechanism Rad9 regulates DNA end resection^13, 14^, even though an antagonistic relationship between Rad9 and the resection-promoting nucleosome remodeller Fun30 has been demonstrated^35, 36^.

Rad9 recruitment to damaged chromatin occurs in all cell cycle phases^19^. However, individual Rad9 recruitment mechanisms are apparently under cell cycle control^17, 33^. Previous data has therefore led to a model where in G1 only one Rad9 recruitment pathway (via interaction with modified nucleosomes, referred to as the ‘histone pathway’^19–22, 25^) is active, while outside of G1 a second Rad9 recruitment pathway (via Dpb11 and 9-1-1, referred to as the ‘Dpb11 pathway’) is additionally available^17, 33^. However, the underlying reason for restricting the Rad9-Dpb11 interaction to specific cell cycle phases is not understood.

Here, we report new aspects in the regulation of Rad9 in the response to DSBs. We find that the Rad9 S/TP sites, which facilitate Dpb11-binding, are also phosphorylated upon DNA damage independently of the cell cycle phase. DNA damage-dependent phosphorylation of these sites can be detected even in G1 cells or upon inhibition of CDK. Notably, these phosphorylation events depend on prior chromatin-recruitment of Rad9 via the ‘histone pathway’ and on the integrity of the SCD domain of Rad9. Furthermore, the Rad9 phosphorylation facilitates the interaction between Rad9 and Dpb11, similarly to our previous results on the CDK-dependent mode of interaction. These findings suggest that Dpb11 and Rad9 can interact even in G1, where Dpb11 is not involved in recruiting Rad9 to damaged chromatin.

## Results

### DNA damage induces phosphorylation of Rad9 S/TP sites and binding of Rad9 to Dpb11

Orthologs of Rad9 and Dpb11 were found to interact in different organisms^17, 18, 29, 37^. In case of budding yeast, our previous work has shown that Rad9 specifically interacts with Dpb11 in cells arrested in M phase, but not in cells arrested in G1^17^. The cell cycle-regulation of the interaction is achieved by CDK-dependent phosphorylation of two S/TP motifs on Rad9 (S462 and T474, referred to as Rad9 S/TP sites hereafter), which are recognized by the BRCT1 + 2 domain of Dpb11^17^.

**Figure 1:**
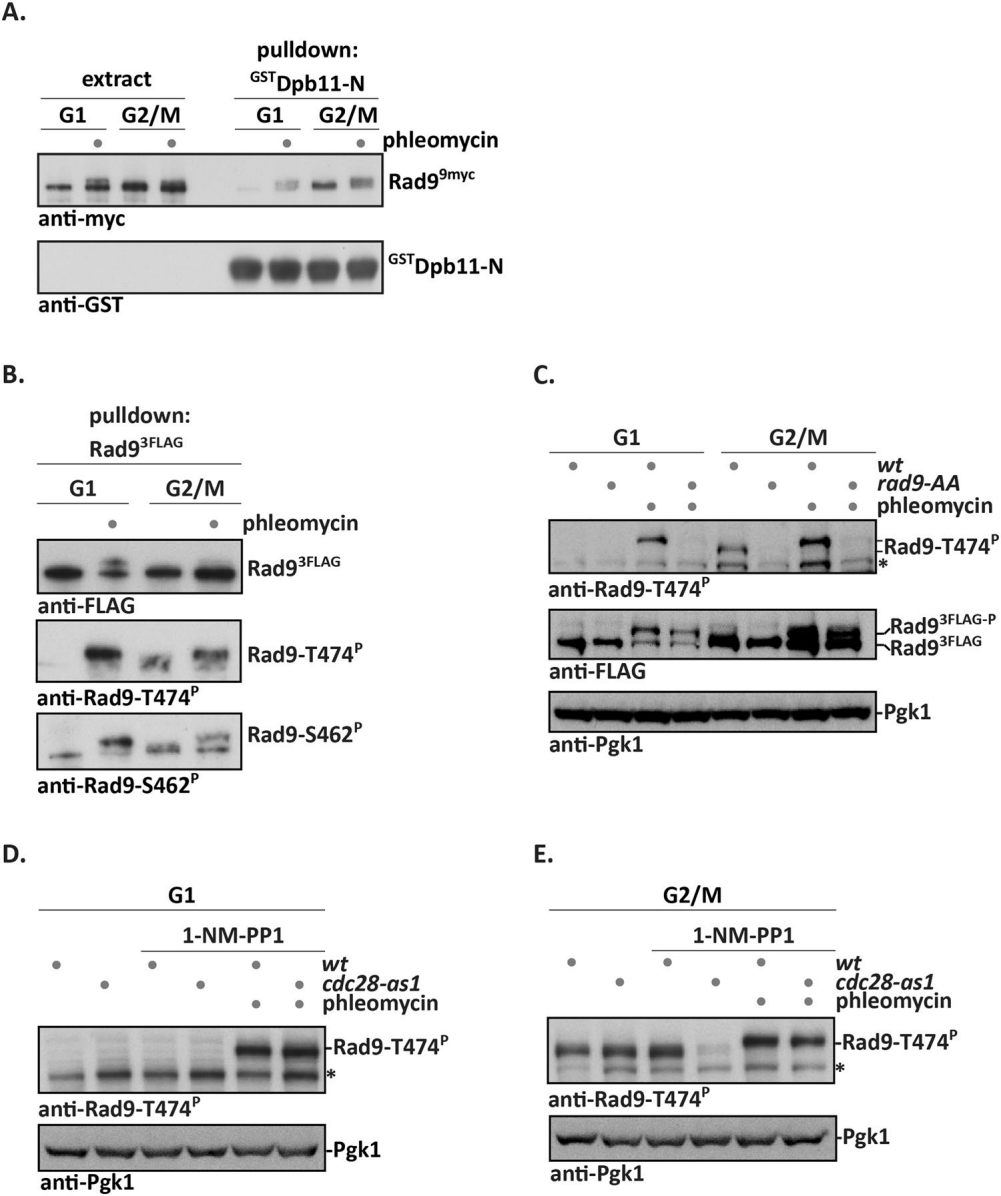
A CDK-independent, DNA damage-dependent mode of Rad9-S462 and -T474 phosphorylation and interaction with Dpb11. (**A**) DNA damage stimulates the Rad9-Dpb11 interaction in cell extracts. GST pulldown experiment with ^GST^Dpb11-N (contains BRCT1 + 2, which is the Rad9 interaction site) and extracts from Rad9^*9myc*^-expressing cells arrested in G1 (α-factor arrest) or M phase (nocodazole arrest) and treated with phleomycin or mock treated. FACS profiles in Fig. S1B. (**B**,**C**) Phosphorylation of Rad9-S462 and -T474 is stimulated by DNA damage in G1. (**B**) Rad9^3FLAG^ was purified from cells treated as in (**A**) by FLAG-IP. Phosphorylation of Rad9 S/TP sites was determined using Rad9-S462p and Rad9-T474p phosphorylation-specific antibodies. FACS profiles in Fig. S1C. **(C)** Cells treated as in **(A)** were used to prepare whole cell extract, which was probed with the Rad9-T474p phosphorylation-specific antibody. The *rad9-AA* strain (harbouring the S462A and T474A mutations) ﻿was used as specificity control. Pgk1 immunoblot serves as loading control. FACS profiles in Fig. S1D. (**D,E**) CDK inhibition does not affect damage-induced Rad9 S/TP phosphorylation. **(D)** 1-NM-PP1 was used to inhibit CDK in G1-arrested *cdc28-as1* cells, but this did not affect Rad9-T474 phosphorylation after DNA damage. FACS profiles in Fig. S1E. (**E**) As in (**D**), but with M phase-arrested cells. 1-NM-PP1 treatment abolished T474 phosphorylation in undamaged *cdc28-as1* cells, demonstrating that CDK is inhibited under these conditions. In contrast T474 is efficiently phosphorylated after phleomycin treatment, even after CDK inhibition. Pgk1 immunoblot serves as loading control. The asterisk denotes a crossreactive band. FACS profiles in Fig. S1F.

We observed that Rad9^*9myc*^ from cell extracts of cells containing MMS-induced DNA damage showed increased interaction with ^GST^Dpb11 in pulldown experiments (Fig. S1A). Strikingly, even when we used cells arrested in G1, we found that DNA damage treatment with the DSB-inducing agent phleomycin resulted in an increased interaction of Rad9^*9myc*^ with ^GST^Dpb11 (Figs 1A and S1B). Phleomycin treatment causes Rad9 to undergo a phospho-shift (Fig. 1A)^8, 10–12^. Notably, we found Dpb11 to associate with this hyperphosphorylated form of Rad9 (Fig. 1A). In contrast, in M phase cell extracts Rad9^*9myc*^ was able to interact with ^GST^Dpb11 even in the absence of DNA damage treatment (Fig. 1A), consistent with our previous result on the CDK regulation of Rad9^17^.

The interaction between Rad9 and Dpb11 critically depends on phosphorylation of S462 and T474 on Rad9^17^. We therefore tested, whether phosphorylation of these sites is also induced by DNA damage. To this end, we used our previously generated phosphorylation-specific antibodies directed against Rad9-epitopes containing either phosphorylated S462 or phosphorylated T474, respectively^17^ (note that anti-Rad9-T474p is highly specific for the phosphorylated form, while anti-Rad9-S462p retains some binding to the unmodified form). When we purified Rad9 via IP from M phase cells, we observed that these Rad9 S/TP sites were phosphorylated in the presence as well as in the absence of DNA damage, consistent with these sites being modified by CDK (Figs 1B and S1C)^17^. Notably, we observed that the S/TP sites were also phosphorylated specifically in phleomycin-treated G1 cells, but not in the absence of DNA damage (Fig. 1B, note the phleomycin-induced phosphorylation shift). The anti-Rad9-T474p antibody can also detect Rad9 S/TP phosphorylation from cell extracts. Figure 1C shows Rad9-T474 phosphorylation in undamaged M phase cells, as well as damaged G1 and M phase cells, but not in undamaged G1 cells, corroborating the result of the IP experiment. Moreover, cells expressing the *rad9-ST462,474AA* variant (referred to as *rad9-AA* hereafter) did not show any reactivity with the Rad9-T474p antibody, confirming specificity (Figs 1C and S1D). We therefore conclude that there are two different modes of Rad9 S/TP phosphorylation: mode 1, which is cell cycle-regulated and depends on CDK^17^, and mode 2, which is DNA damage-dependent.

In order to verify that the DNA damage-induced phosphorylation of Rad9 in G1 is CDK-independent, we used a *cdc28-as1* mutant strain, in which CDK activity was effectively inhibited by addition of 1-NM-PP1, but this did not abrogate Rad9-T474 phosphorylation after DNA damage (Figs 1D and S1E). We furthermore used the same strategy of CDK-inhibition in M phase-arrested cells and found that CDK-dependent phosphorylation of Rad9-T474 in undamaged cells was effectively inhibited in line with previous results (Figs 1E and S1F)^17^. Notably, phleomycin treatment efficiently stimulated phosphorylation of Rad9-T474 in M phase-arrested cells after CDK inhibition (Fig. 1E). Taken together, these data show that the damage-induced phosphorylation of the Rad9 S/TP sites occurs independently of the cell cycle phase and CDK activity (Fig. 1E).

### DNA damage-induced phosphorylation of the Rad9 S/TP sites depends on the apical checkpoint kinases Mec1 and Tel1 and the Rad9 SCD

**Figure 2:**
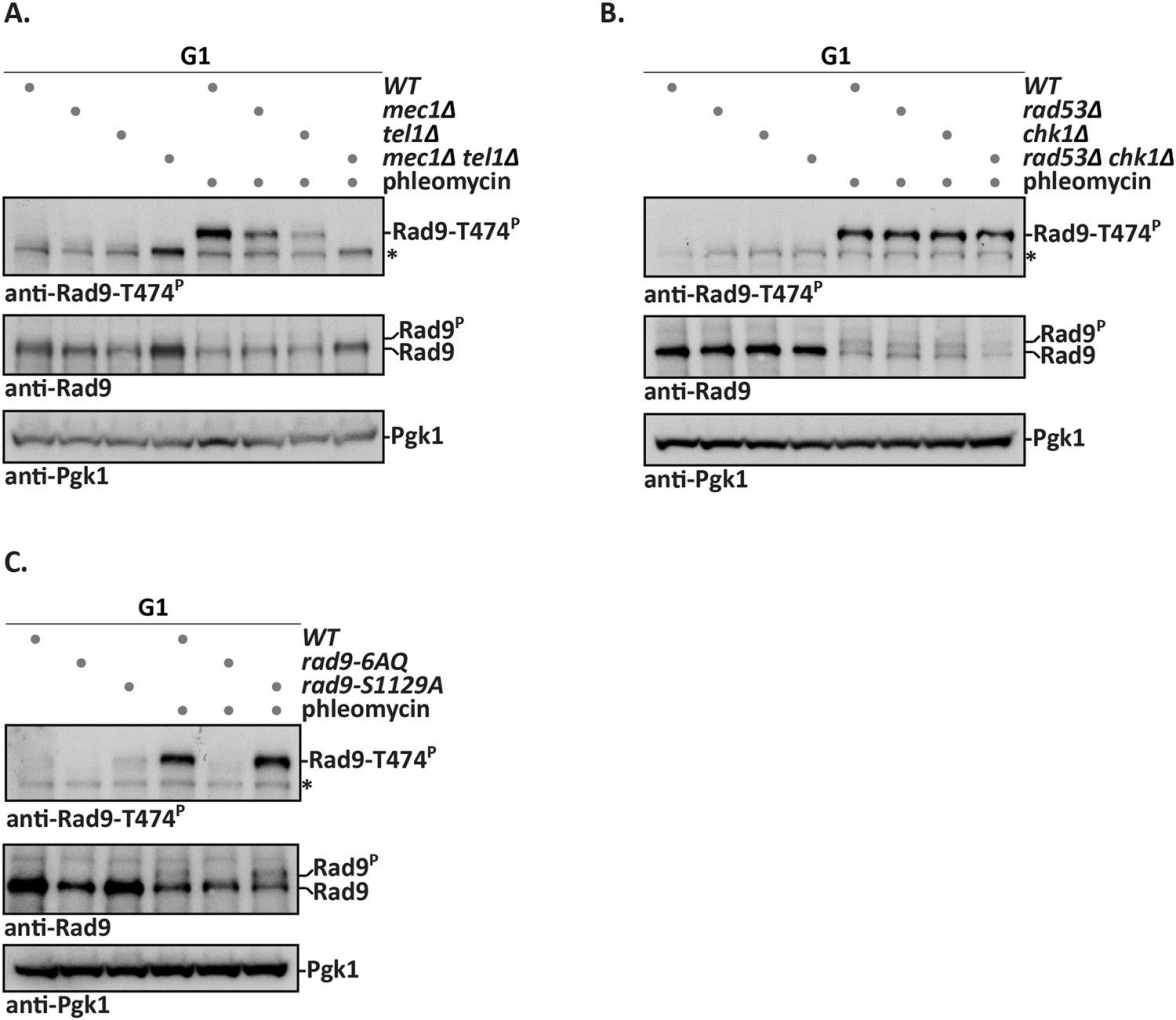
Mec1 and Tel1 are required for phosphorylation of Rad9 S/TP sites after DNA damage. (**A**) Rad9-T474 phosphorylation after DNA damage depends on the apical checkpoint kinases Mec1 and Tel1. G1-arrested cells with indicated genotypes were treated with phleomycin, Rad9-T474 phosphorylation was visualized by immunoblotting. Strains containing the *mec1Δ* mutation are in *sml1Δ* background. Pgk1 immunoblot serves as loading control. An asterisk denotes a crossreactive band. FACS profiles in Fig. S2A. (**B**) Rad9-T474 phosphorylation after DNA damage is independent of checkpoint effector kinases Chk1 and Rad53. G1-arrested cells with indicated genotypes were treated with phleomycin and subjected to analysis with immunoblots as in (**A**). Strains containing the *rad53Δ* mutation are in *sml1Δ* background. FACS profiles in Fig. S2B. (**C**) Integrity of the Rad9 SCD domain is important for damage-induced Rad9 S/TP phosphorylation. Treatment and immunoblotting of *WT*, *rad9-6AQ* and *rad9-S1129A* strains as in (**A**). FACS profiles in Fig. S2C.

Upon DNA damage, the apical checkpoint kinases Mec1 and Tel1 target several sites on Rad9^8, 11, 12^. Therefore, we tested whether also the phosphorylation of Rad9 S/TP sites would be dependent on Mec1 and Tel1. Notably, T474 phosphorylation in G1-arrested, phleomycin-treated cells was reduced in *mec1Δ* and *tel1Δ* mutant cells and completely abolished in a *mec1Δ tel1Δ* double mutant (Figs 2A and S2A). Therefore, phosphorylation of Rad9 S/TP sites shows a dependency on the apical checkpoint kinases, which is highly similar to overall damage-induced Rad9 phosphorylation (indicated by the phosphoshift, Fig. 2A). In contrast, the deletion mutants of the checkpoint effector kinases *RAD53* or *CHK1*, alone or in combination, did not affect T474 phosphorylation (Figs 2B and S2B).

It could thus be reasoned that Rad9 S/TP sites are themselves targeted by the apical checkpoint kinases Mec1 and Tel1, similarly to Rad9 S/TQ sites^8, 11, 12^. However, we did not obtain evidence that purified Mec1 would show activity towards Rad9 S/TP sites *in vitro* (data not shown). Therefore, we considered the option that the apical checkpoint kinases could promote Rad9 S/TP site phosphorylation indirectly. Possible mechanisms include a priming role of Rad9 S/TQ phosphorylation or Mec1/Tel1 promoting chromatin recruitment of a factor involved in S/TP site phosphorylation, such as the kinase acting on Rad9 or Rad9 itself (via γH2A). Indeed, we found that a Rad9 mutant harbouring six S/T to A exchanges in the S/TQ cluster domain (SCD) (*rad9-6AQ*)^12^ abolished phleomycin-induced phosphorylation of Rad9 S/TP sites in G1 (Figs 2C and S2C). In contrast, CDK-dependent phosphorylation of these sites in M phase was unaffected by the *rad9-6AQ* mutant (Fig. S2D). Previous work has suggested that phosphorylation of the SCD would induce Rad9 dimerization^38^. However, we excluded dimerization as underlying cause for the SCD-dependency, as the dimerization-defective Rad9-S1129A variant^38^ showed normal phosphorylation of Rad9-T474 both in G1 after DNA damage and in M phase (Figs 2C and S2D). Overall, we conclude that Mec1/Tel1-dependent phosphorylation of the SCD of Rad9 is required for phosphorylation of the Rad9 S/TP sites upon DNA damage, but additional direct and/or indirect roles of the apical checkpoint kinases are possible.

### Chromatin-recruitment of Rad9 is required for phosphorylation of the Rad9 S/TP sites

Previous studies suggest two possible pathways by which Rad9 is recruited to damaged chromatin (‘histone pathway’^19–22, 25^ and ‘Dpb11 pathway’^17, 33^). In G1 cells, however, the ‘histone pathway’ is apparently uniquely required^17, 33^. Given our findings, we re-investigated the possibility that the ‘Dpb11 pathway’ may be contributing to Rad9 recruitment and also tested the alternative model that the damage-induced Rad9-Dpb11 interaction in G1 may rely on the ‘histone pathway’.

**Figure 3:**
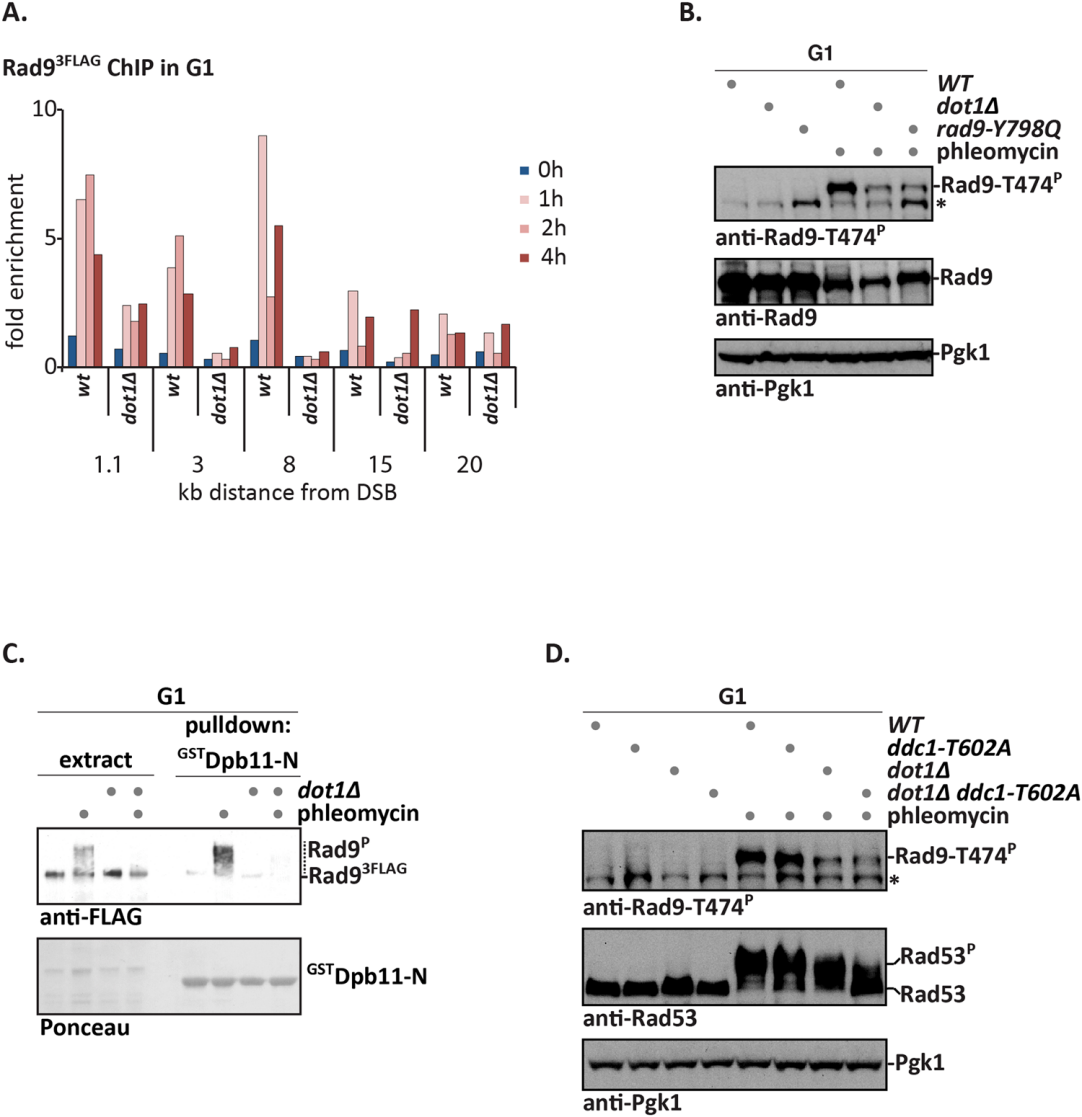
Dot1 is required for phosphorylation of Rad9 S/TP sites and interaction with Dpb11. (**A**) Dot1 is required for Rad9 association with a double strand break (DSB). Induction of an non-repairable DSB at *MAT* locus using galactose-induced HO. ChIP against Rad9^3FLAG^ to regions from 1.1 kb to 8 kb distal of the DSB site and 1, 2 and 4 h after DSB induction. FACS profiles in Fig. S3A. (**B**–**D**) The ‘histone pathway’ is required for efficient damage-induced phosphorylation of Rad9-T474 and binding to Dpb11. (**B**) Phleomycin-induced T474 phosphorylation is reduced in *dot1Δ* or *rad9-Y789Q* cells (deficient in TUDOR domain-dependent binding to K79-methylated H3). Experiment as in Fig. 2A, but with *WT*, *dot1Δ* and *rad9-Y789Q* cells. Pgk1 immunoblot serves as loading control. An asterisk denotes a crossreactive band. FACS profiles in Fig. S3D. (**C**) Dpb11 does not bind to Rad9 from extracts of G1-arrested, phleomycin-treated *dot1Δ* cells. GST-Dpb11-N pulldown as in Fig. 1A (**D**) DNA damage-induced Rad9-T474 phosphorylation in G1 as in (**B**), but with *WT, ddc1-T602A, dot1Δ* or *dot1Δ ddc1-T602A* strains. FACS profiles in Fig. S3E.

A critical element of the ‘histone pathway’ is K79-methylation of H3, which is catalysed by the Dot1 methyltransferase^23^ and recognized by the TUDOR domain of Rad9^19, 22^. We therefore tested Rad9 binding to damaged chromatin by ChIP in G1-arrested cells and used the GAL-HO system to induce a site-specific, non-repairable DSB at the *MAT* locus^39^. While Rad9 became enriched in the chromatin region surrounding the DSB in *WT* cells after DSB induction, Rad9 enrichment was strongly decreased in *dot1Δ* cells (Figs 3A and S3A). Consistent with a lack of Rad9 recruitment to damaged chromatin, we observed that damage-induced phosphorylation of Rad9 S/TP sites was reduced in G1 cells lacking Dot1 (Figs 3B and S3D).

Intriguingly, deletion of *DOT1* caused a strong reduction of Rad9-T474 phosphorylation in phleomycin-treated G1 cells (Fig. 3B). To ascertain that this effect originated from a defect in the interaction of Rad9 with nucleosomes (i.e. a deficient ‘histone pathway’), we introduced the corresponding H3 K79-binding-defective mutation in the Rad9 TUDOR domain (*rad9-Y798Q* ^19^) and observed a highly similar reduction in Rad9-T474 phosphorylation in this background (Fig. 3B). This effect was again specific for the DNA damage-induced phosphorylation of Rad9 S/TP sites (mode 2), as neither a *dot1Δ* nor a *rad9-Y798Q* mutation diminished CDK-dependent phosphorylation of Rad9-T474 in M phase (Fig. S3B,C).

We expected that a lack of Rad9 S/TP phosphorylation would translate into an inability to bind to Dpb11. Indeed, we observed a reduced association of Rad9 in ^GST^Dpb11 pulldowns in the absence of Dot1, when the Rad9-Dpb11 association was induced by phleomycin-treatment of G1-arrested cells (Fig. 3C).

We observed that *dot1Δ* as well as *rad9-Y798Q* cells showed minor residual Rad9-T474 phosphorylation in G1 (Fig. 3B), which responded dose-dependently to phleomycin (Fig. S3D). Since M phase cells could compensate a defect in the ‘histone pathway’ by Dpb11-dependent Rad9 recruitment (‘Dpb11 pathway’^17, 33^), we tested if the ‘Dpb11 pathway’ would be responsible for the residual phosphorylation of Rad9. However, we did not observe any additional defect in Rad9-T474 phosphorylation, when we introduced the Dpb11-binding-deficient *ddc1-T602A* allele either alone or in combination with *dot1Δ* (Figs 3D and S3E). Therefore, we conclude that Rad9 S/TP site phosphorylation after DNA damage as well as the interaction of Dpb11 and Rad9 are dependent on the ‘histone pathway’.

### Forced Rad9 recruitment to damaged chromatin allows efficient Rad9 S/TP site phosphorylation

The ‘histone pathway’ facilitates Rad9 recruitment to damaged chromatin. We reasoned that the dependency of the damage-induced Rad9 S/TP-phosphorylation on the ‘histone pathway’ could be easily explained, if Rad9 needed to localize to damaged chromatin in order to become phosphorylated. We therefore aimed to create a cellular scenario, which forces Rad9 localization to damaged chromatin independently of the ‘histone pathway’.

**Figure 4:**
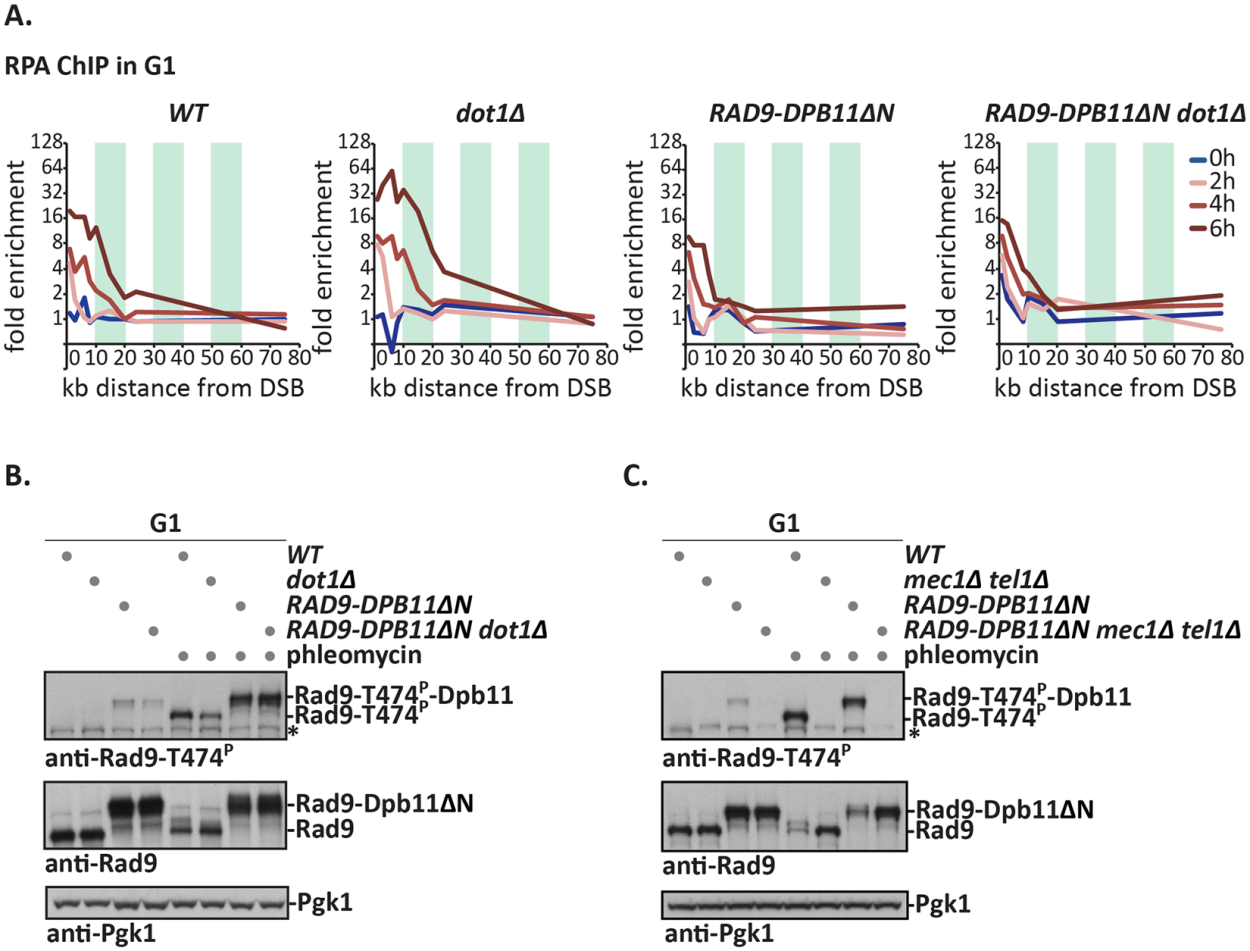
A Rad9-Dpb11 fusion forces Rad9 recruitment to DSBs and T474 phosphorylation independently of the ‘histone pathway’. (**A**) The Rad9-Dpb11 fusion blocks resection, also in the absence of Dot1. RPA-ChIP at the indicated positions from an HO-induced DSB (0, 2, 4 and 6 h after HO induction) in *WT*, *dot1Δ, RAD9-DPB11ΔN* and *RAD9-DPB11ΔN dot1Δ* indicates the extent of DNA end resection. FACS profiles in Fig. S4A. (**B**,**C**) The Rad9-Dpb11 fusion bypasses the requirement for Dot1, but not for Mec1 and Tel1. Measurement of Rad9-T474 phosphorylation as in Fig. 2A, but in G1-arrested cells expressing the Rad9-Dpb11 fusion in (**B**) *WT* and *dot1Δ* background or (**C**) *WT* and *mec1Δ tel1Δ* background. Immunoblotting against Rad9 or Rad9-T474 phosphorylation. A Pgk1 immunoblot serves as loading control. An asterisk denotes a crossreactive band. FACS profiles in Fig. S4C and E respectively. Strains containing the *mec1Δ* mutation are in *sml1Δ* background.

We have previously shown that covalent protein-fusions containing the BRCT3 + 4 domain of Dpb11 localized efficiently and cell cycle-independently to damaged chromatin^36^. In case of Rad9, this fusion protein (Rad9-Dpb11∆N, referred to as Rad9-Dpb11 fusion) hyperactivates DNA damage checkpoint signalling^17^. To ascertain that this fusion acts by forcing Rad9 localization to damaged chromatin, we measured inhibition of DNA end resection by Rad9 as a read-out of Rad9 function^13, 14^. Therefore, we tested the extent of resection at an HO-induced DSB using ChIP against the ssDNA-binding protein RPA. In the presence of the Rad9-Dpb11 fusion, the spreading of resection was strongly reduced independently of the cell cycle phase and the functionality of the ‘histone pathway’ (Figs 4A and S3A,B). These data therefore suggest a model whereby the Rad9-Dpb11 fusion forces enhanced Rad9 recruitment to damaged chromatin, where it causes hyperactivation of the DNA damage checkpoint, as well as inhibition to DNA end resection, consistent with previous results^17, 40^.

Next, we used the Rad9-Dpb11 fusion to test its effects on Rad9 S/TP site phosphorylation. We found that after DNA damage induction Rad9-T474 phosphorylation was enhanced in the context of the Rad9-Dpb11 fusion and even present to low levels without induction of exogenous damage (Figs 4B and S4C,D). Importantly, in the context of the fusion Rad9-T474 phosphorylation was largely independent of Dot1 (Fig. 4B), while it still showed dependency on the apical kinases Mec1 and Tel1 (Figs 4C and S4E). Overall, these data suggest that the function of the ‘histone pathway’ in damage-induced Rad9 S/TP phosphorylation lies entirely in the recruitment of Rad9 to damaged chromatin.

### Rad9 S/TP phosphorylation in G1 is dispensable for DNA end resection and the DNA damage checkpoint

Outside of G1, CDK-phosphorylation of Rad9 S/TP sites provides a pathway of Rad9 recruitment to damaged chromatin^17^. However, in case of the damage-induced Rad9 phosphorylation mode, our data rather suggest a function downstream of recruitment (Figs 3 and 4). So far, Rad9 is known to have two functions – (A) inhibition of DNA end resection and (B) activation of the DNA damage checkpoint. Therefore, we tested if the *rad9-AA* variant would show a G1-specific defect in any of these functions.

**Figure 5:**
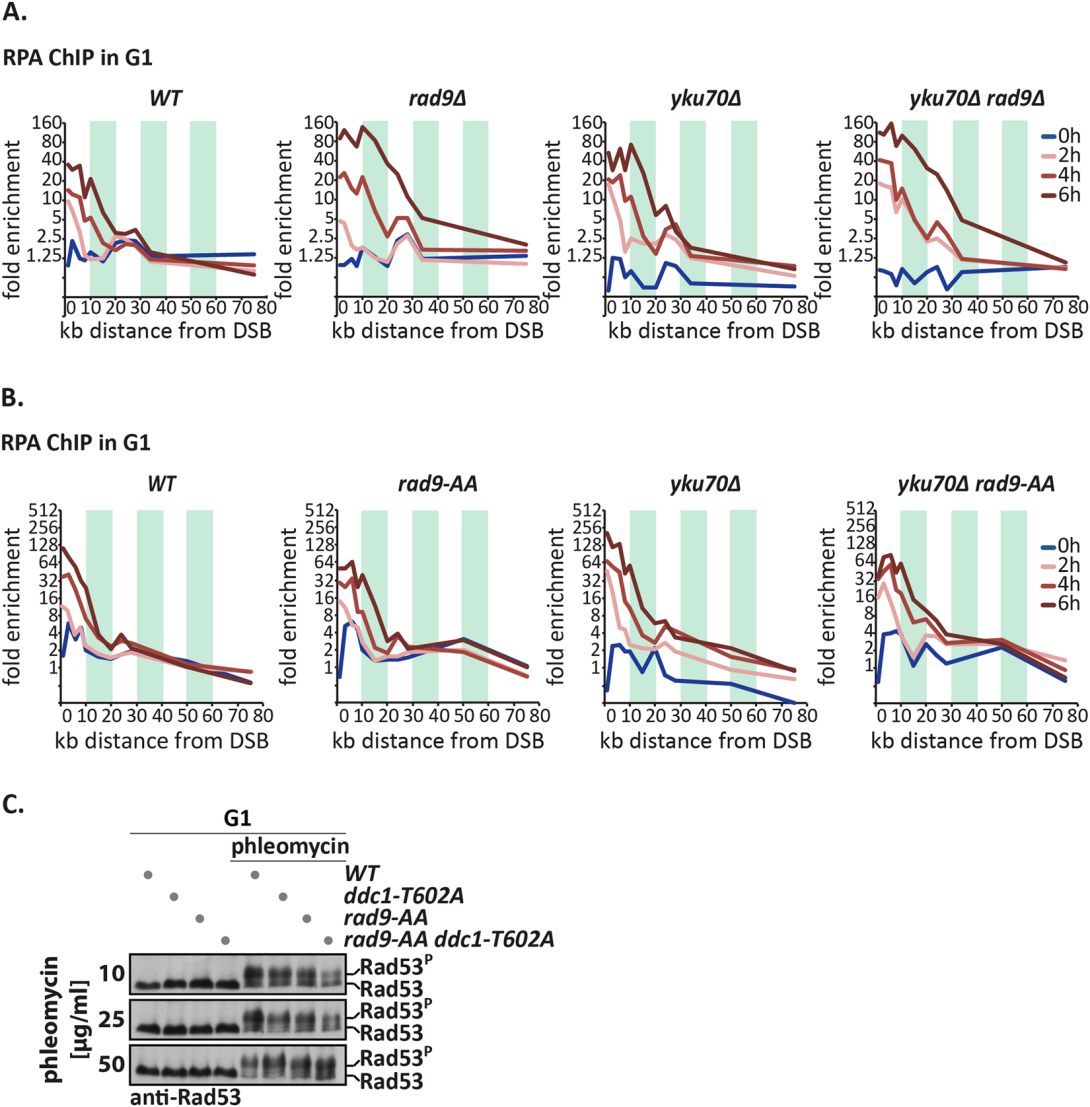
Lack of damage-induced Rad9 S/TP phosphorylation does not directly affect checkpoint signalling or DNA end resection. (**A**,**B**) The *rad9-AA* mutant – in contrast to the *rad9Δ* mutant – does not induce hyper-resection in G1-arrested cells. A site-specific DSB was induced at the *MAT* locus using galactose-induced HO in G1-arrested cells. DNA end resection is shown by ChIP against RPA at 0, 2, 4 and 6 h after HO induction within 0–80 kb distance to the DSB. (**A**) Resection was measured in *WT*, *rad9Δ*, *yku70Δ* and *rad9Δ yku70Δ* strains. FACS profiles in Fig. S5A. (**B**) as (**A**), but with *WT*, *rad9-AA*, *yku70Δ* and *rad9-AA yku70Δ* strains. FACS profiles in Fig. S5B. (**C**) The *rad9-AA* mutant does not induce apparent defects in checkpoint activation in G1 even in ﻿the﻿ background of the *ddc1-T602A* mutation. Hyperphosphorylation of Rad53 induced by different concentrations of phleomycin added to the growth medium is used as measure of checkpoint activation. FACS profiles in Fig. S5C.

To measure DNA end resection, we again used the GAL-HO system and ChIP against RPA. Consistent with previous studies^13, 14^, we observed enhanced spreading of the RPA-ChIP signal away from the site of the DSB in *rad9Δ* and *dot1Δ* strains, indicating enhanced DNA end resection in the absence of chromatin-bound Rad9 (Figs 5A and S5A). However, we did not observe any significant change in DNA end resection in G1-arrested *rad9-AA* cells, even in the absence of Yku70, suggesting that the Rad9-Dpb11 interaction on its own is not required for regulation of DNA end resection in G1 (Figs 5A,B and S5B).

For checkpoint activation, we have previously shown that the *rad9-AA* mutant on its own does not induce any defects in the phosphorylation of the Rad53 effector kinase in G1 cells^17^ (see also Figs 5C and S5C). We therefore considered the possibility that a defect in damage-induced Rad9 S/TP phosphorylation may be compensated by other factors. Specifically, we tested compensation by the 9-1-1 complex, since both Rad9 and 9-1-1 could in principle serve to recruit Dpb11 to sites of DNA damage. Therefore, we combined the *rad9-AA* mutant with the *ddc1-T602A* mutant, which abolishes the 9-1-1-Dpb11 interaction. However, while the *ddc1-T602A* mutation strongly reduced Dpb11 association with a site-specific DSB in G1-arrested cells, the *rad9-AA* mutant did not induce a measurable defect (Fig. S5D). Consistently, checkpoint activation was still largely functional in the *rad9-AA* mutant, even in the *ddc1-T602A* background (Fig. 5C).

Overall, the functional relevance of the damage-induced mode of Rad9 S/TP phosphorylation therefore remains unclear. Given the high degree of redundancy in the checkpoint signalling network, it is highly likely that a defect in the *rad9-AA* mutant is compensated, perhaps by phosphorylation of an additional factor or by other phosphorylation sites in Rad9.

## Discussion

S/TP site phosphorylation has been shown to be an important cellular mechanism that facilitates cell cycle controls (see ref. 41 for a review on control of the DNA damage response by S/TP phosphorylation). Our study provides experimental evidence for DNA damage-dependent, but cell cycle-independent phosphorylation of the budding yeast checkpoint protein Rad9 at S/TP sites. These sites have previously been shown to be phosphorylated by CDK and to facilitate interaction with Dpb11^17, 42^. Notably, we found that also the DNA damage-induced, CDK-independent phosphorylation of Rad9 leads to an interaction with Dpb11. When testing the attributes of DNA damage-induced phosphorylation, we found that it requires the histone methyltransferase Dot1, indicating a dependency on the ‘histone pathway’, which is known to target Rad9 to damaged chromatin^19–22, 25^. Moreover, the covalent Rad9-Dpb11 fusion, which is known to tether Rad9 to damaged chromatin^17, 40^, bypasses this dependency on the ‘histone pathway’.

We found that damage-induced Rad9 S/TP phosphorylation is abolished, when Rad9 cannot be recruited to damaged chromatin. In this regard, damage-induced Rad9 S/TP phosphorylation is highly similar to Rad9 S/TQ phosphorylation^19–23^, which can be measured as an overall Rad9 phosphorylation shift. Conversely, we observed that forced localization of Rad9 to chromatin, reinstates S/TP phosphorylation, suggesting that Rad9 has to be recruited to damaged chromatin in order to become phosphorylated for both damage-induced S/TP phosphorylation and S/TQ phosphorylation.

Our data therefore suggest that Rad9 S/TP sites are targeted by a chromatin-localized kinase. The apical checkpoint kinases Mec1 and Tel1 would fulfil this requirement, as they are specifically active at damaged chromatin^43^. Consistently, we found that damage-induced Rad9 S/TP phosphorylation is abolished in a *mec1Δ tel1Δ* double mutant. However, this influence could also be indirect, since Mec1 and Tel1 are necessary for efficient phosphorylation of the Rad9 SCD, which itself is required for damage-induced phosphorylation of Rad9 S/TP sites. Moreover, we could not find any *in vitro* evidence to support that Mec1 or Tel1 would directly target S/TP motifs. Currently, the best candidates for this novel mode of Rad9 S/TP phosphorylation, are the transcriptional kinases of the CDK family – Kin28, Srb10, Bur1 and Ctk1 – given their similarity to Cdc28 and their chromatin-localization. In the future, it will therefore be interesting to test the connection between transcriptional CDKs and the DNA damage checkpoint.

Several studies have collectively suggested a model of cell cycle-regulated Rad9 recruitment and activation in budding yeast^17, 33, 42^ and fission yeast^4^. These models suggest that the ‘histone pathway’ is exclusively required for Rad9 recruitment to damaged chromatin in G1, while in M phase both ‘histone pathway’ and ‘Dpb11 pathway’ are active. While our study suggests that Rad9 and Dpb11 can interact in G1 as well, this view of Rad9 recruitment pathways is not affected, since Rad9 recruitment via the ‘histone pathway’ is upstream of and required for damage-induced Rad9 S/TP site phosphorylation and the Rad9-Dpb11 interaction in G1. Dpb11, therefore, does apparently not function as Rad9 recruiter in G1. As such, it is currently unresolved what function the damage-induced phosphorylation of Rad9 S/TP sites and subsequent binding to Dpb11 could have. We have not found any phenotypes in the G1 checkpoint or in the control of DNA end resection in G1, when we used the *rad9-AA* mutant. So far, we have investigated possible redundancies in Dpb11 recruitment (using the *ddc1-T602A* allele, Fig. 5) and Rad9 recruitment (using the *dot1Δ* allele, ref. 17), but also this did not reveal a defect. Therefore, the damage-induced Rad9 phosphorylation at S/TP sites may either act redundantly with a currently unknown factor or mediate an entirely new function.

Eukaryotic orthologs of Rad9 have been shown to be recruited to damaged chromatin by related mechanisms^4, 27–31, 44–47^. Specifically, both fission yeast Crb2 and human 53BP1 were found to interact with the respective Dpb11 orthologs^29, 37^. Notably, in human cells 53BP1 and TOPBP1 were found to interact specifically in G1^37^. This interaction, therefore, does seemingly not require CDK-phosphorylation, but would rather be consistent with a DNA damage-induced mode of interaction as described here. The phosphorylation sites on 53BP1 that mediate TOPBP1-binding are currently unknown and it remains to be established whether the DNA damage-induced mode of the Rad9-Dpb11 interaction is evolutionary conserved.

Given the abundance of target proteins that are modified at S/TP sites by CDK^48^, S/TP site phosphorylation is often interpreted as phosphorylation by CDK. Our results caution, however, that this may be an oversimplified view. It will be interesting to see if CDK-independent S/TP site phosphorylation is a general phenomenon that can be observed on other proteins as well. Phosphoproteomic experiments in human cells treated with etoposide or γ-irradiation have rather suggested an opposite trend, as S/TP phosphorylation was generally decreased^49^. However, this decrease is caused by the inhibition of Cdk1 and Cdk2 after DNA damage in human cells. In order to test whether a substantial number of S/TP phosphorylation substrates become modified specifically after DNA damage, a system would be required, where CDK is not generally downregulated after DNA damage. While budding yeast fulfils this requirement, previous phosphoproteomic studies of the DNA damage response in budding yeast have primarily focussed on damage-induced S/TQ phosphorylation and checkpoint kinase dependencies^50, 51^. A systematic investigation of DNA damage-induced S/TP phosphorylation, as well as the involved kinases, therefore appears worthwhile.

## Methods

### Materials

All yeast strains used in this study were derived from W303 MATa and were constructed using standard methods^52^. Cells were grown in YP glucose or YP raffinose media at 30 °C. All strains used in this study are listed in Supplementary Table 1, all antibodies in Supplementary Table 2.

### Measurement of Rad9 and Rad53 phosphorylation

Cells were grown in YP glucose media at 30 °C or 24 °C. Cell cycle synchronization was performed using α-factor (5 μg/ml or 0.25 μg/ml for *bar1Δ* mutants) or nocodazole (5 μg/ml) for 2–3 hours. To inhibit CDK, a strain containing the *cdc28-as1* allele^53^ was treated with 1 µM 1-NM-PP1. To induce DNA damage, phleomycin (Invivogen) was added to the medium to a final concentration of 50 µg/ml - or concentrations as indicated. Denaturing cell extracts were prepared by alkaline lysis follo﻿wed by trichloroacetic acid (TCA) precipitation and precipitated proteins were collected by centrifugation and resuspended in SDS-PAGE sample buffer containing 8 M urea for subsequent SDS-PAGE analysis.

To detect Rad9 phosphorylation on S462 and T474, previously described phospho-specific antibodies were used^17^. Rad53 phospho-shifts were resolved on 10% acrylamide gels.

### Rad9 Immunoprecipitations

For Rad9^3FLAG^ IPs cell extracts were prepared from 200 OD yeast cells treated as above for cell cycle arrest and DNA damage. Cells were harvested, washed in ice-cold sorbitol buffer (1 M sorbitol, 25 mM Hepes pH 7.6), and resuspended in a 1:1 ratio with lysis buffer supplemented with protease and phosphatase inhibitors (100 mM Hepes, 200 mM KOAc, 0.1% NP-40, 10% glycerol, 2 mM β-mecaptoethanol, 100 nM okadaic acid, 10 mM NaF, 20 mM β-glycerophosphate, 400 μM PMSF, 4 μM aprotinin, 4 mM benzamidin, 400 μM leupeptin, 300 μM pepstatin A), snap-frozen to liquid nitrogen and lysed using a Spex Sample Prep cryo mill. The extracts were cleared by centrifugation and incubated with anti‐FLAG agarose resin (Sigma) for 1 hour (4 °C, rotation). After five washes with lysis buffer, Rad9^3FLAG^ was eluted twice with 0.5 mg/ml 3xFLAG peptide (Sigma). The elutions were pooled and proteins were precipitated with TCA prior to analysis on 4–12% NuPAGE gels (Invitrogen) and standard western blotting.

### GST-Dpb11 pulldowns

The Dpb11-Rad9 interaction was tested as described^17^. GST, GST-Dpb11 FL or a GST-Dpb11 fragment containing BRCT1 + 2 were immobilized on glutathione sepharose 4B (GE Healthcare) and incubated with 600 ml ammonium sulphate-precipitated (57%) cell extracts (in 200 mM KOAc, 100 mM Hepes pH 7.6, 10% glycerol, 0.02% NP-40, 2 mM β-mercaptoethanol, 20 mM β-glycerophosphate, 10 mM NaF, 100 mM okadaic acid, protease inhibitors) corresponding to 50 OD yeast cells. The pulldown was incubated 1 hour (4 °C, rotation), washed and eluted by boiling in SDS-PAGE sample buffer.

### Chromatin Immunoprecipitation (ChIP) to a DSB and qPCR analysis

For chromatin immunoprecipitation of Rad9, RPA and Dpb11, cells were grown in YP raffinose to an OD of 0.5 and cell cycle arrest was induced with α-factor or nocodazole. A single double strand break at the *MAT* locus was introduced by inducing the HO endonuclease from the galactose promoter by addition of galactose to the cultures to a 2% final concentration. 100 ODs of cells were crosslinked with formaldehyde (final 1%) for 16 minutes at timepoints as indicated and the reaction was quenched with glycine. Cells were harvested by centrifugation, washed in ice-cold PBS and snap-frozen. Cell pellets were resuspended in 800 μl lysis buffer (50 mM HEPES KOH pH 7.5, 150 mM NaCl, 1 mM EDTA, 1% Triton X-100, 0.1% Na-deoxycholate, 0.1% SDS) and lysed with zirconia beads using a bead beating device. The chromatin was sonified to shear the DNA to a size of 200–500 bp. The obtained extracts were cleared by centrifugation, 1% was taken as input sample and 40% were incubated with either anti-FLAG-M2 magnetic beads (Sigma) for 2 hours (Rad9^3FLAG^ ChIPs) or with anti-RPA antibody (AS07-214, Agrisera) followed by 30 min with Dynabeads ProteinA (Invitrogen, for RPA ChIPs). The beads were washed 3x in lysis buffer, 2x in lysis buffer with 500 mM NaCl, 2x in wash buffer (10 mM Tris-Cl pH 8.0, 0.25 M LiCl, 1 mM EDTA, 0.5% NP-40, 0.5% Na-deoxycholate) and 2x in TE pH 8.0. DNA-protein complexes were eluted in 1% SDS, proteins were removed via proteinase K digestion (3 h, 42 °C) and crosslinks were reversed (8 h or overnight, 65 °C). The DNA was subsequently purified using phenol-chloroform extraction and ethanol precipitation and quantified by quantitative PCR (Roche LightCycler480 System, KAPA SYBR FAST 2x qPCR Master Mix, KAPA Biosystems) at indicated positions with respect to the DNA double strand break. As a control, 2-3 control regions on other chromosomes were quantified.

## Electronic supplementary material

Supplementary Info
