# Supplementary material for: A cell cycle-independent mode of the Rad9-Dpb11 interaction is induced by DNA damage
Source: Sci Rep. 2017 Sep 14;7:11650. doi: 10.1038/s41598-017-11937-z (PMC5599684; doi:10.1038/s41598-017-11937-z)
Supplement: Supplementary file 1 — Supplementary Info [file 41598_2017_11937_MOESM1_ESM.pdf]

Supplementary material

A cell cycle-independent mode of the Rad9-Dpb11 interaction is induced by DNA damage

Giulia di Cicco, Susanne C. S. Bantele, Karl-Uwe Reusswig, and Boris Pfander

- **Supplementary figures S1-S14**
- **Supplementary figure legends**
- **Supplementary tables S1-S2**

Figure S1

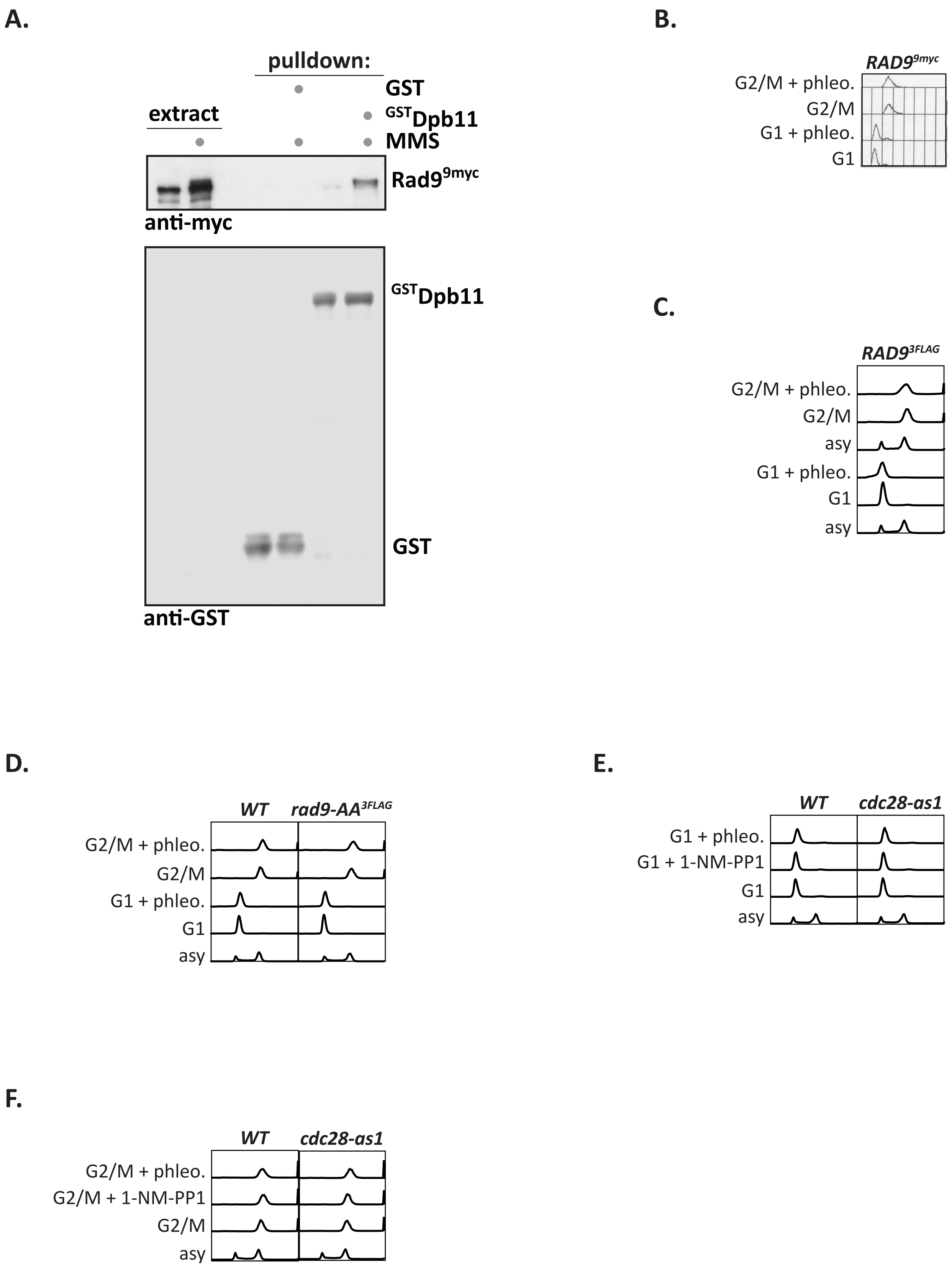
A.
pulldown:
extract
GST
GSTDpb11
MMS
Rad9 9myc
anti-myc
GSTDpb11
GST
anti-GST
B.
RAD9 9myc
G2/M + phleo.
G2/M
G1 + phleo.
G1
C.
RAD9 3FLAG
G2/M + phleo.
G2/M
asy
G1 + phleo.
G1
asy
D.
WT
rad9-AA 3FLAG
G2/M + phleo.
G2/M
G1 + phleo.
G1
asy
E.
WT
cdc28-as1
G1 + phleo.
G1 + 1-NM-PP1
G1
asy
F.
WT
cdc28-as1
G2/M + phleo.
G2/M + 1-NM-PP1
G2/M
asy

Figure S2

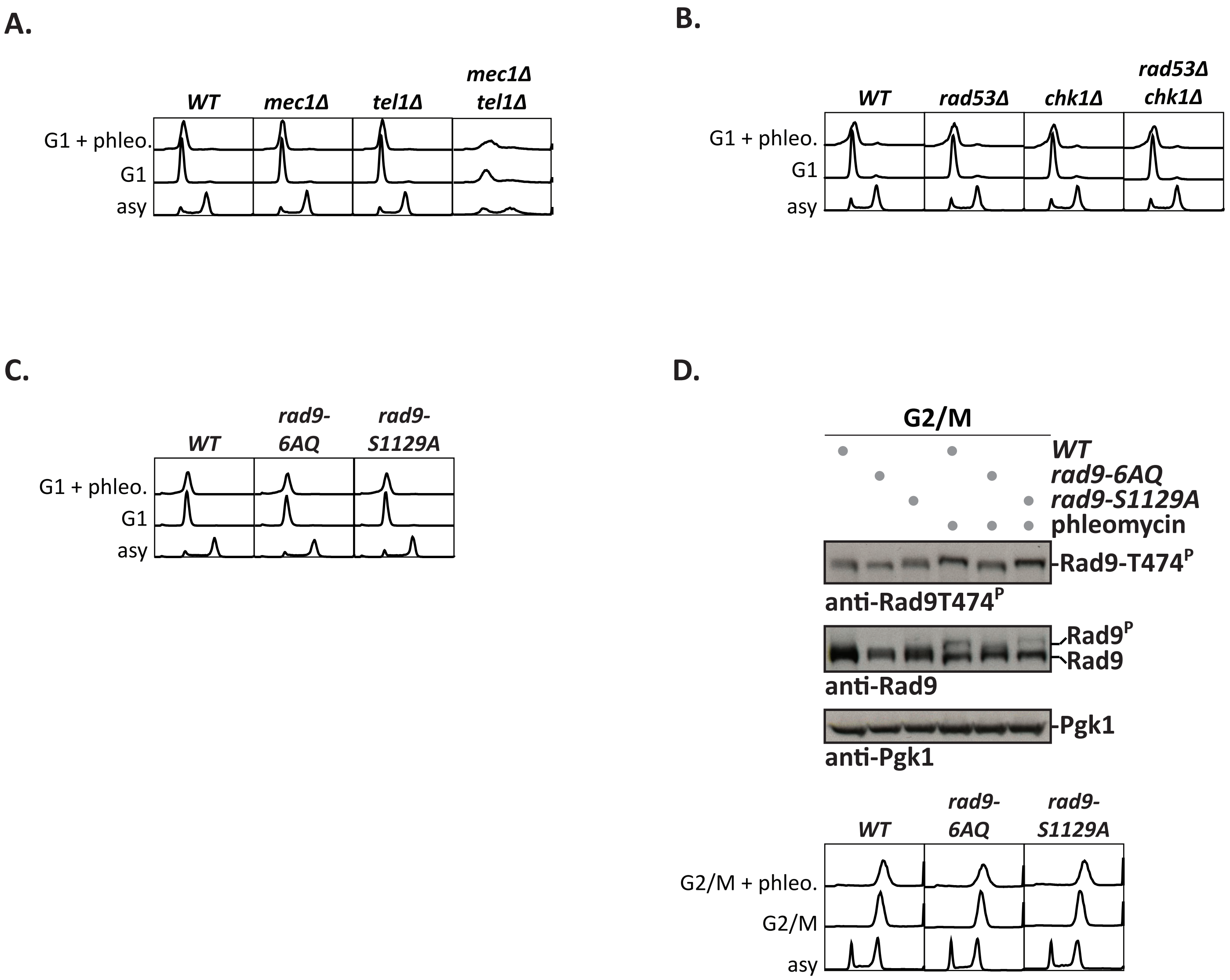

A.
WT
mec1Δ
tel1Δ
mec1Δ tel1Δ
G1 + phleo.
G1
asy
B.
WT
rad53Δ
chk1Δ
rad53Δ chk1Δ
G1 + phleo.
G1
asy
C.
WT
rad9-6AQ
rad9-S1129A
G1 + phleo.
G1
asy
D.
G2/M
WT
rad9-6AQ
rad9-S1129A
phleomycin
Rad9-T474P
anti-Rad9T474P
Rad9P
Rad9
anti-Rad9
Pgk1
anti-Pgk1
WT
rad9-6AQ
rad9-S1129A
G2/M + phleo.
G2/M
asy

Figure S3

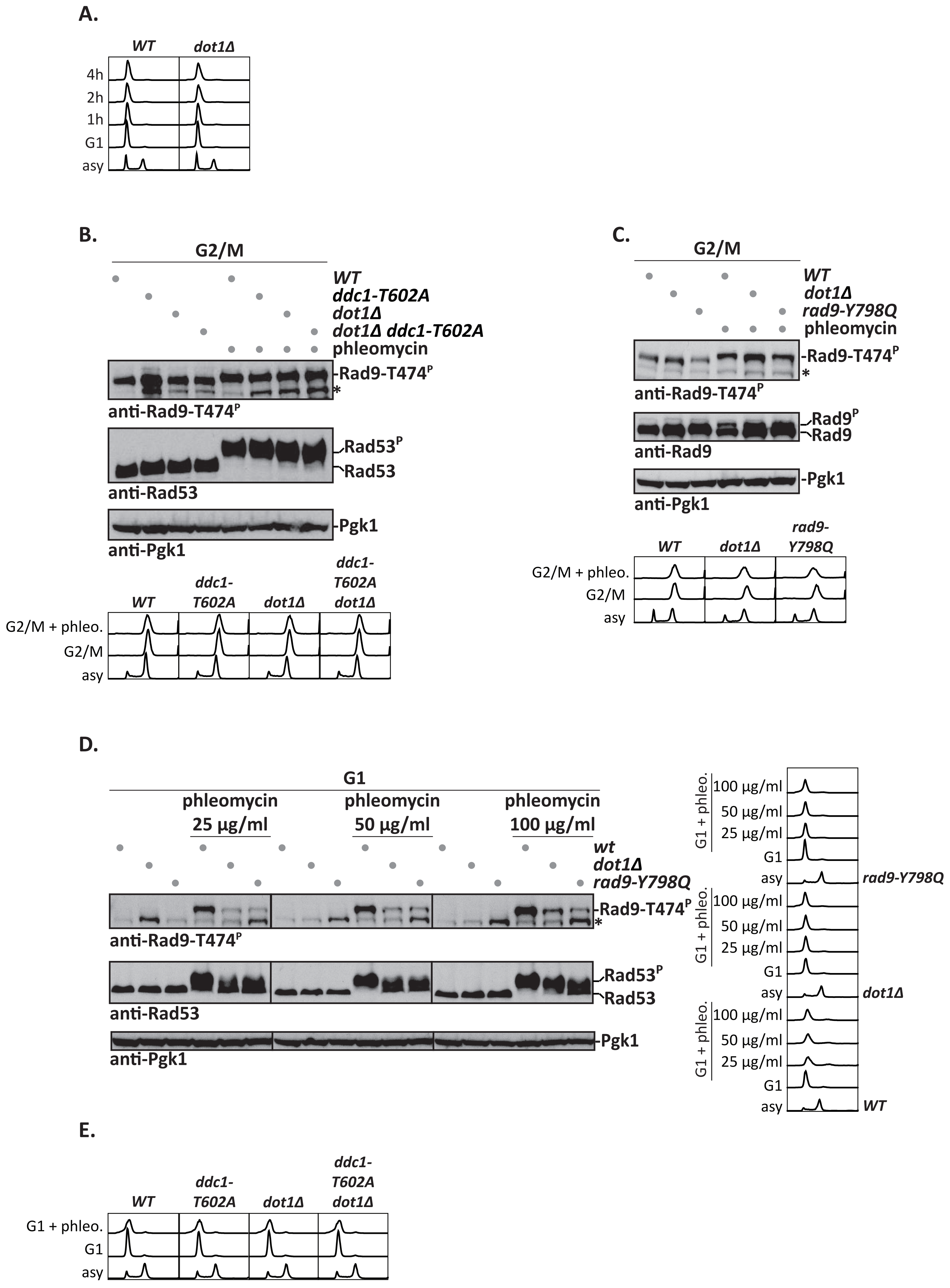
A.
WT
dot1Δ
4h
2h
1h
G1
asy
B.
G2/M
WT
ddc1-T602A
dot1Δ
dot1Δ ddc1-T602A
phleomycin
-Rad9-T474P
*
anti-Rad9-T474P
Rad53P
Rad53
anti-Rad53
-Pgk1
anti-Pgk1
WT
ddc1-T602A
dot1Δ
ddc1-T602A dot1Δ
G2/M + phleo.
G2/M
asy
C.
G2/M
WT
dot1Δ
rad9-Y798Q
phleomycin
-Rad9-T474P
*
anti-Rad9-T474P
Rad9P
Rad9
anti-Rad9
-Pgk1
anti-Pgk1
WT
dot1Δ
rad9-Y798Q
G2/M + phleo.
G2/M
asy
D.
G1
phleomycin 25 µg/ml
phleomycin 50 µg/ml
phleomycin 100 µg/ml
wt
dot1Δ
rad9-Y798Q
-Rad9-T474P
*
anti-Rad9-T474P
Rad53P
Rad53
anti-Rad53
-Pgk1
anti-Pgk1
G1 + phleo.
100 µg/ml
50 µg/ml
25 µg/ml
G1
asy
rad9-Y798Q
dot1Δ
WT
E.
WT
ddc1-T602A
dot1Δ
ddc1-T602A dot1Δ
G1 + phleo.
G1
asy

Figure S4

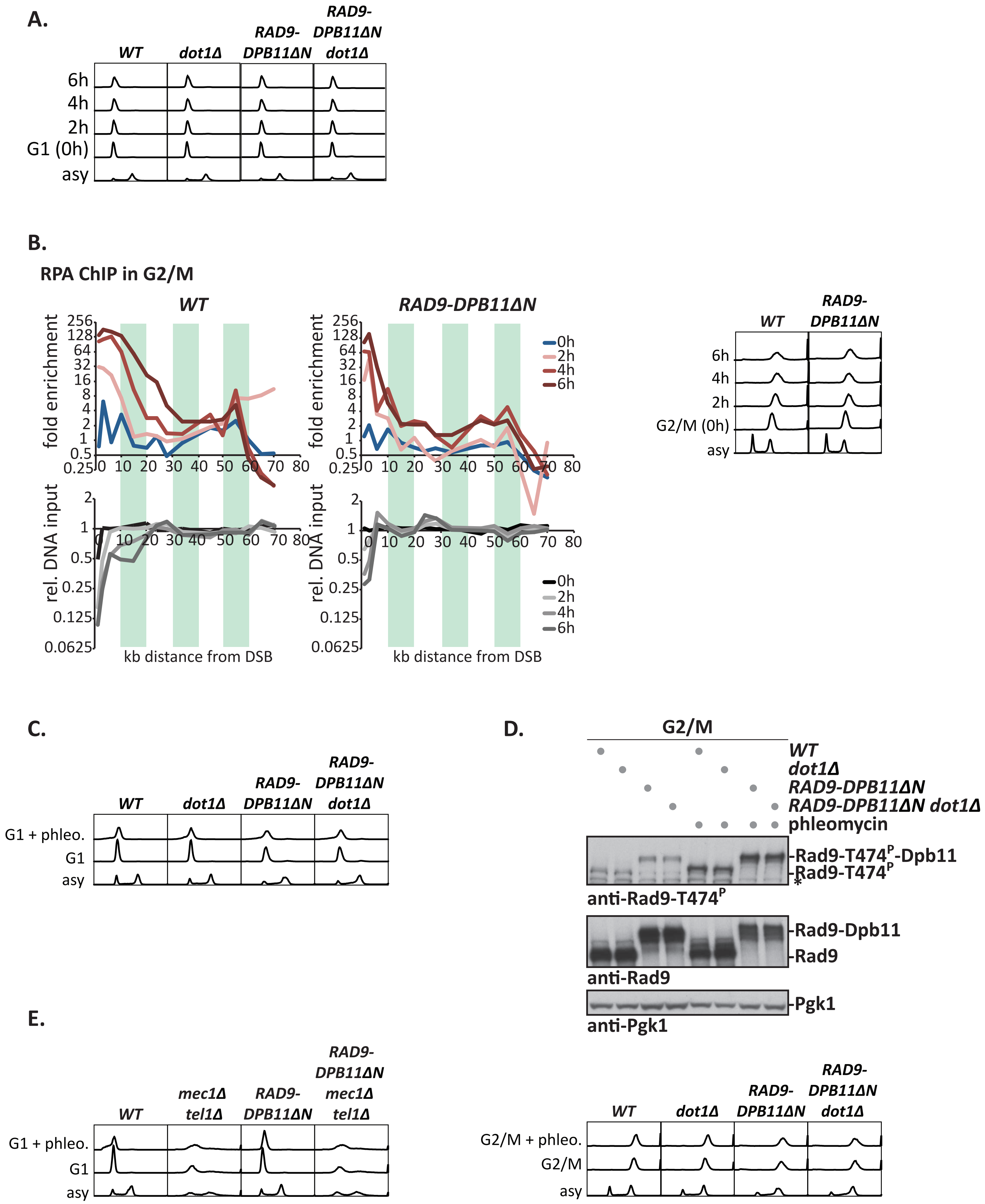
A.
WT
dot1Δ
RAD9-DPB11ΔN
RAD9-DPB11ΔN dot1Δ
6h
4h
2h
G1 (0h)
asy
B.
RPA ChIP in G2/M
WT
RAD9-DPB11ΔN
fold enrichment
256
128
64
32
16
8
4
2
1
0.5
0.25
0 10 20 30 40 50 60 70 80
0h
2h
4h
6h
rel. DNA input
2
1
0.5
0.25
0.125
0.0625
kb distance from DSB
G2/M (0h)
C.
G1 + phleo.
G1
D.
G2/M
phleomycin
Rad9-T474^P-Dpb11
Rad9-T474^P
*
anti-Rad9-T474^P
Rad9-Dpb11
Rad9
anti-Rad9
Pgk1
anti-Pgk1
E.
mec1Δ tel1Δ
RAD9-DPB11ΔN mec1Δ tel1Δ
G2/M + phleo.
G2/M

Figure S5

A.

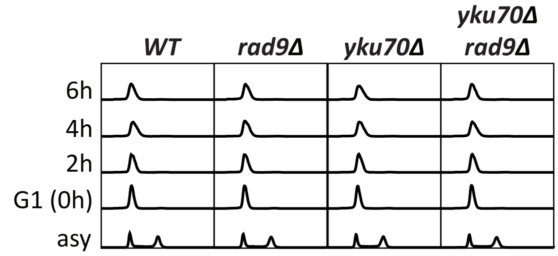

B.

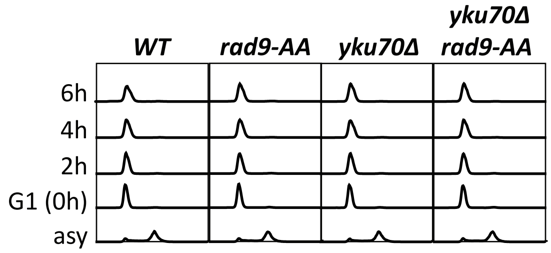

C.

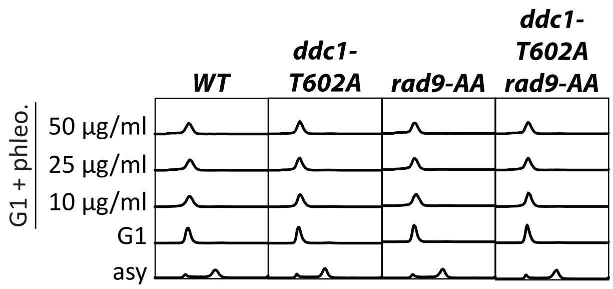

D.

Dpb11^{3FLAG} ChIP in G1

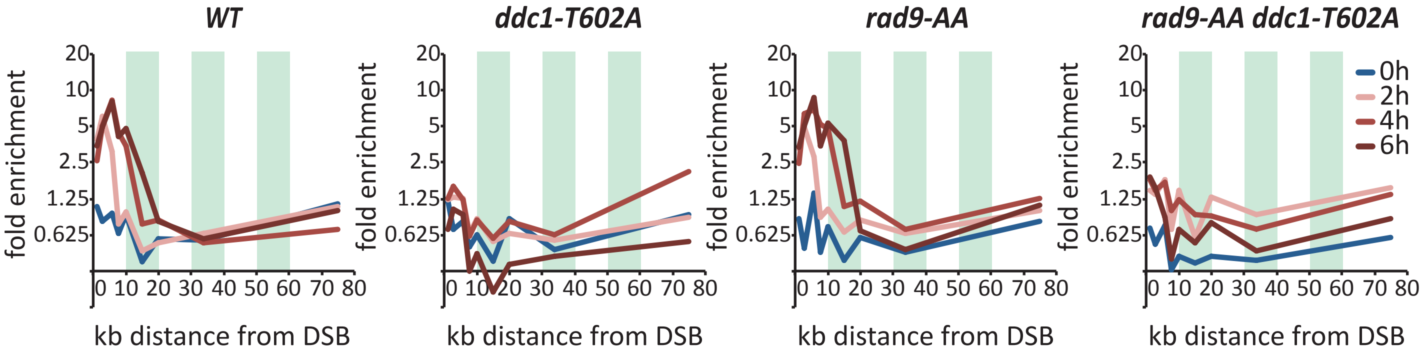

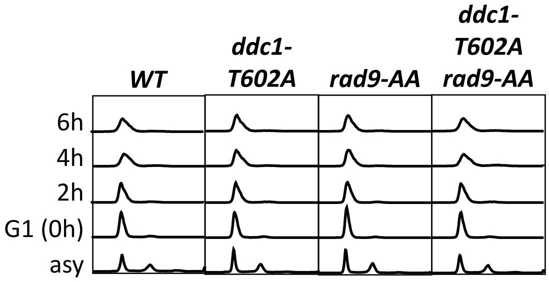

Figure S6

A. relates to Fig. 1A

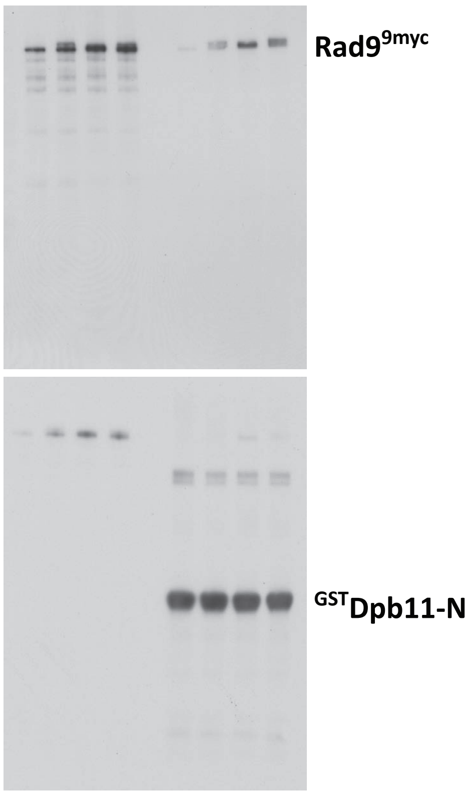

B. relates to Fig. 1B

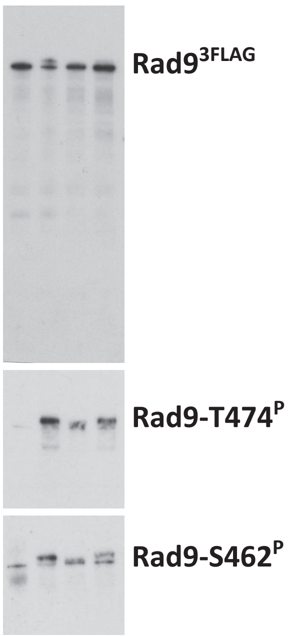

C. relates to Fig. 1C

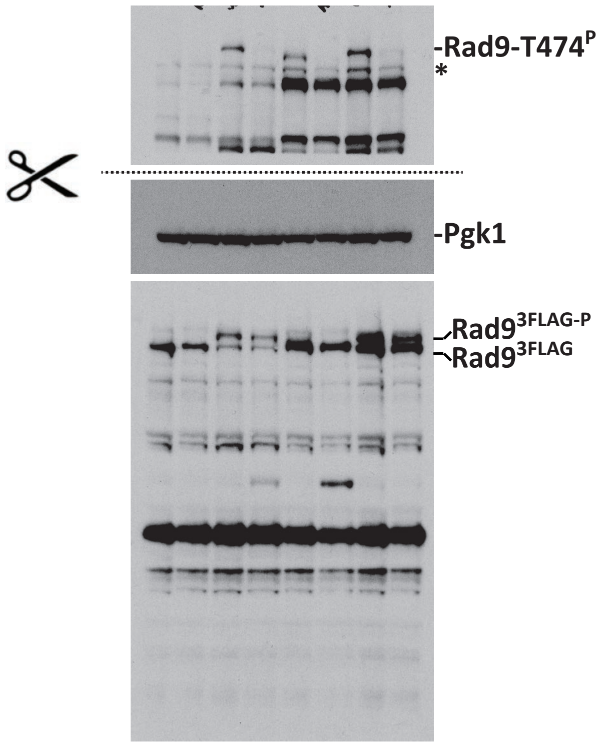

D. relates to Fig. 1D

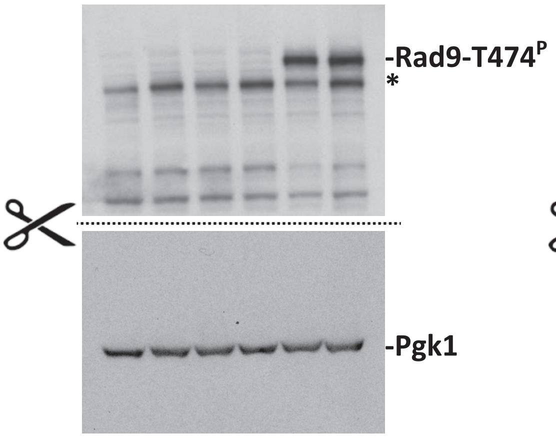

E. relates to Fig. 1E

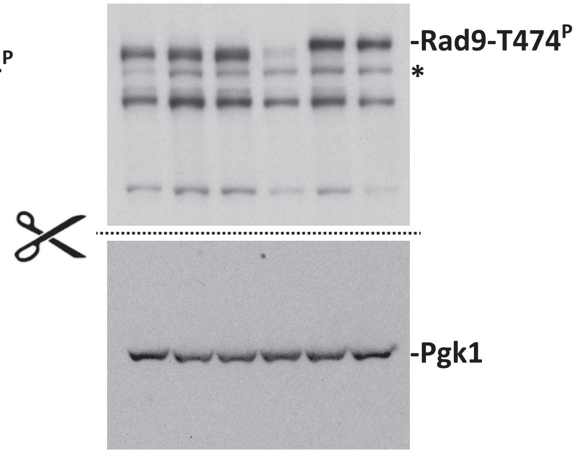

Figure S7

A. relates to Fig. S1A

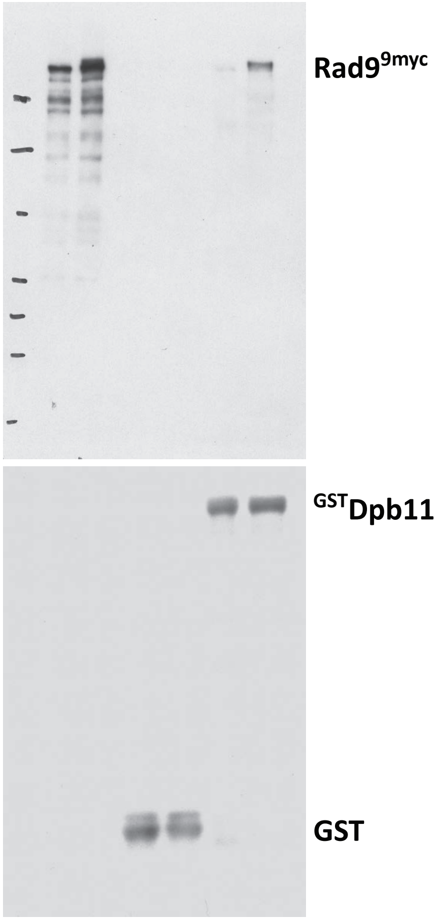

Figure S8

A. relates to Fig. 2A

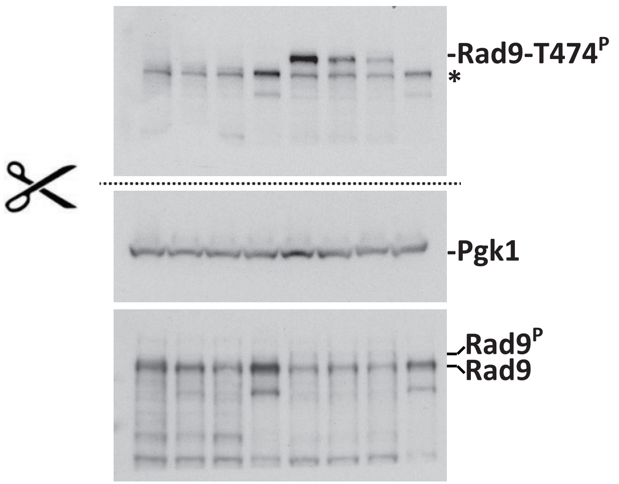

B. relates to Fig. 2B

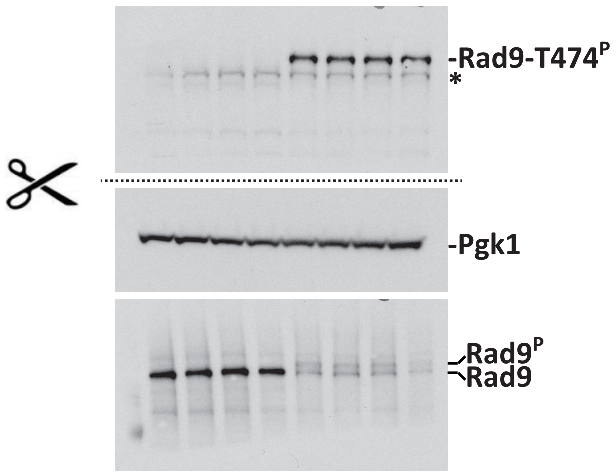

C. relates to Fig. 2C

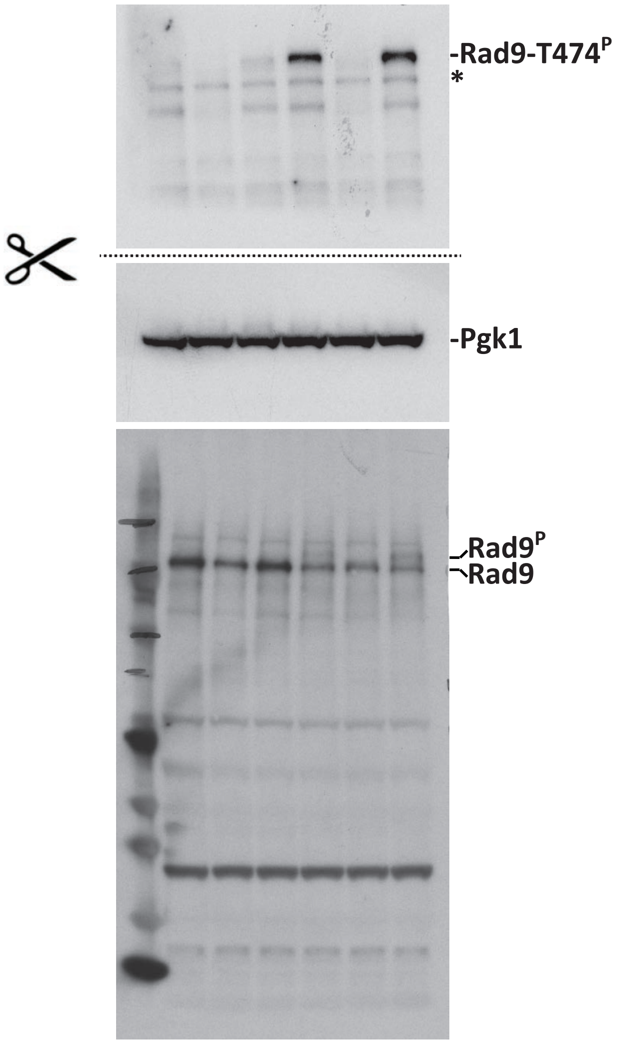

Figure S9

A. relates to Fig. S2D

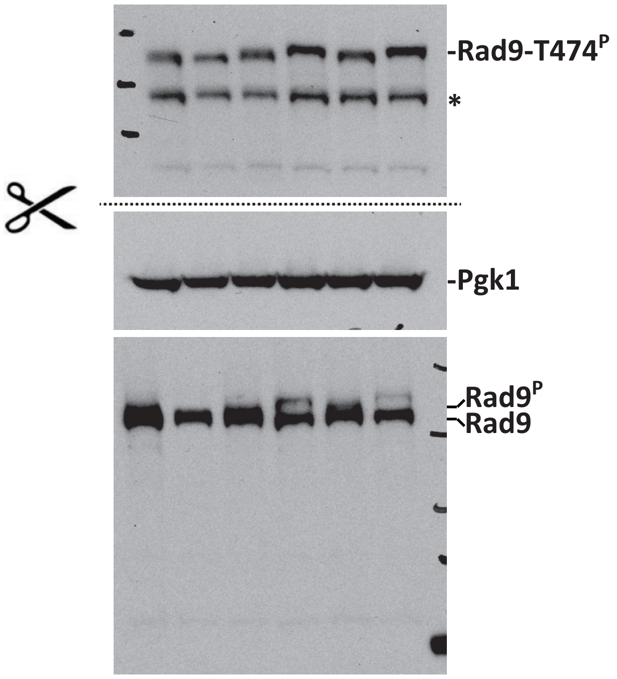

Figure S10

A. relates to Fig. 3B

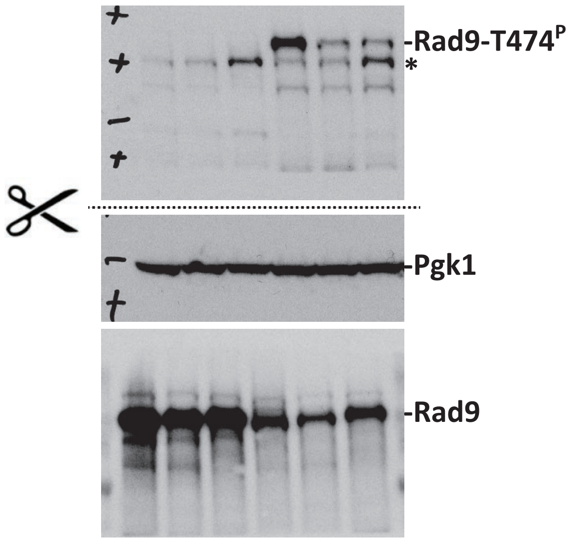

B. relates to Fig. 3C

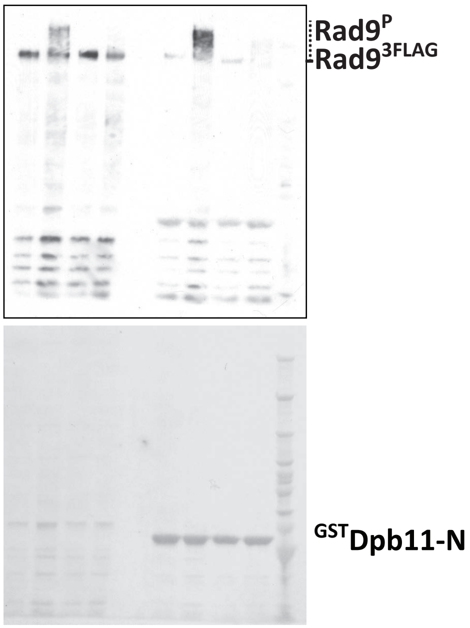

C. relates to Fig. 3D

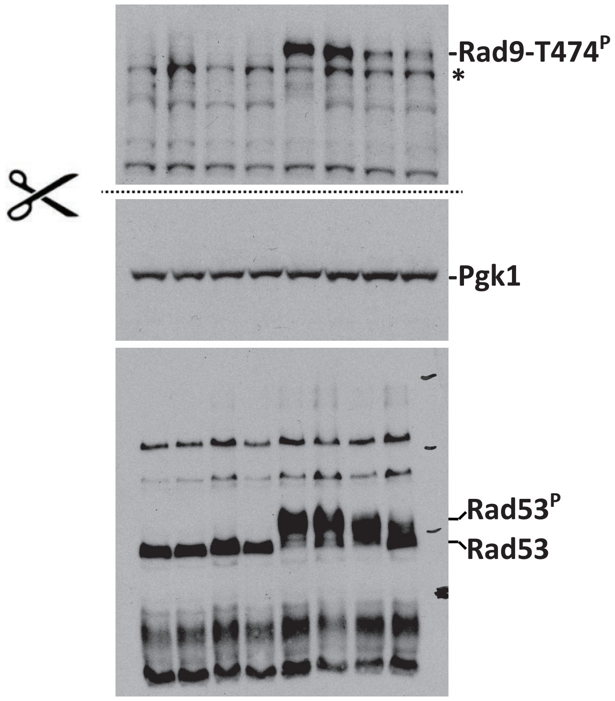

Figure S11

A. relates to Fig. S3B

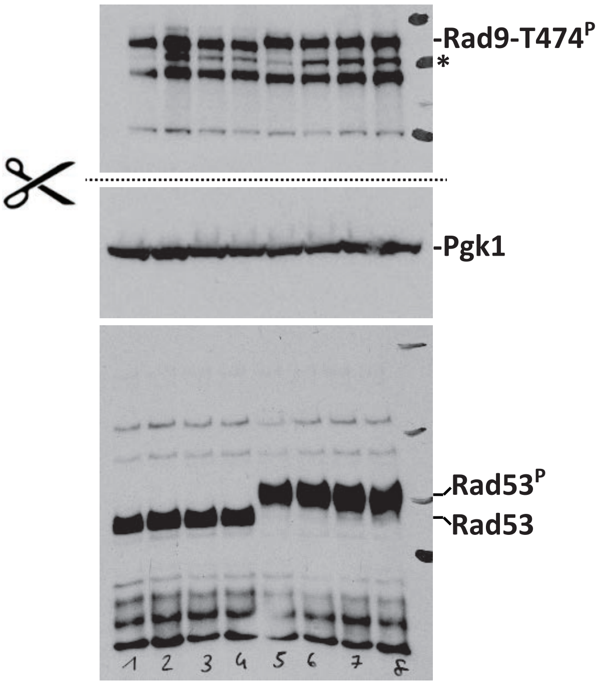

B. relates to Fig. S3C

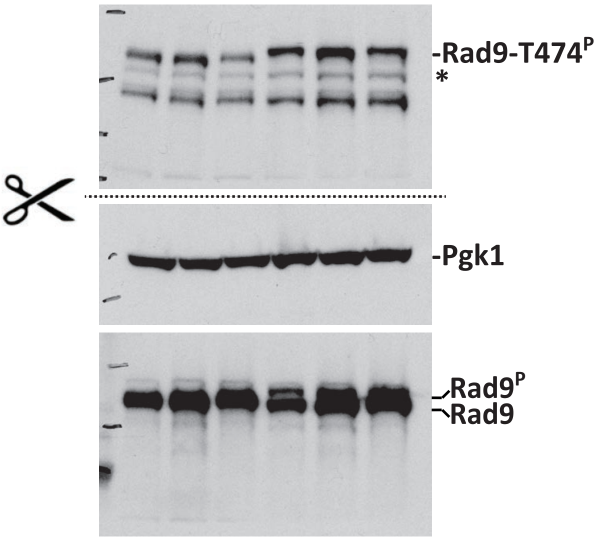

C. relates to Fig. S3D

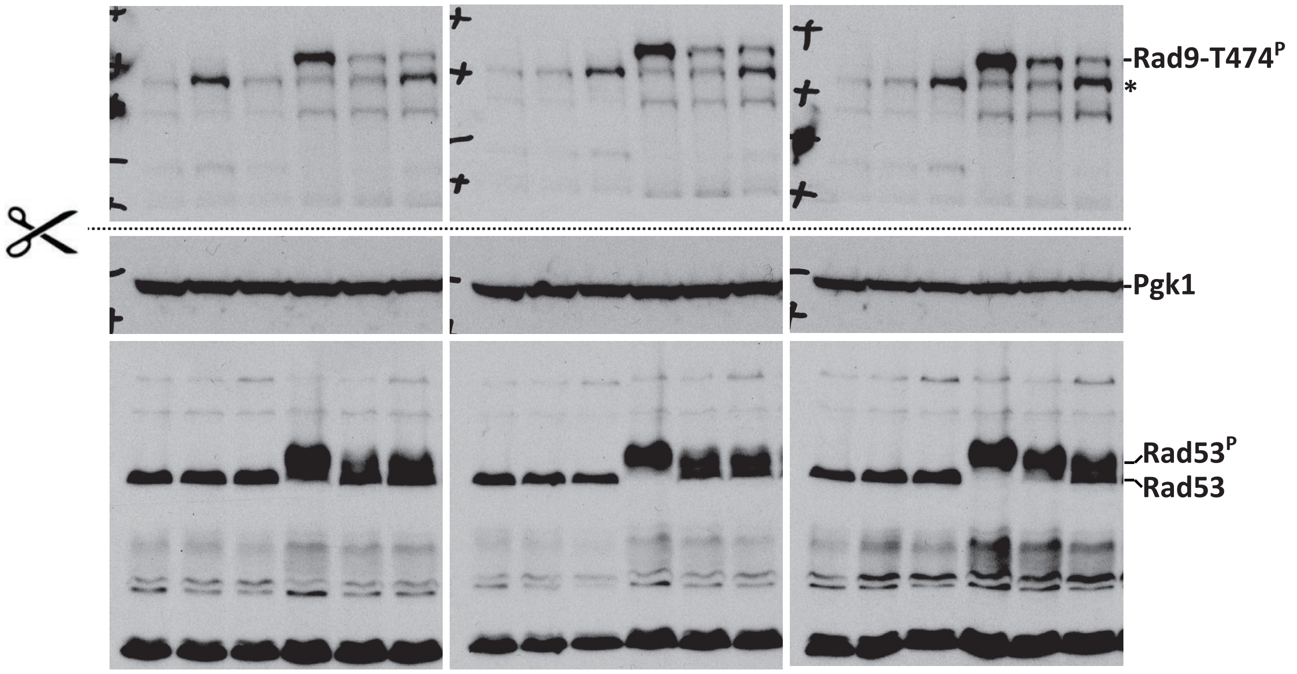

Figure S12

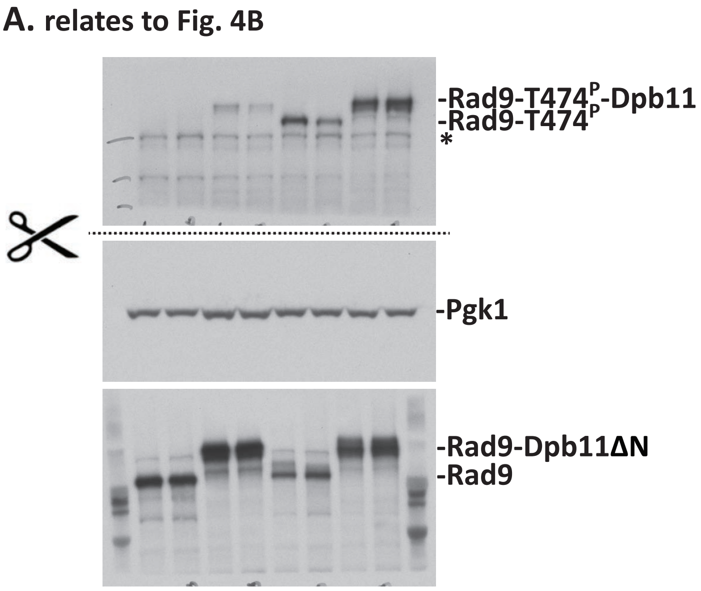

A. relates to Fig. 4B
-Rad9-T474P-Dpb11
-Rad9-T474P
*
-Pgk1
-Rad9-Dpb11ΔN
-Rad9

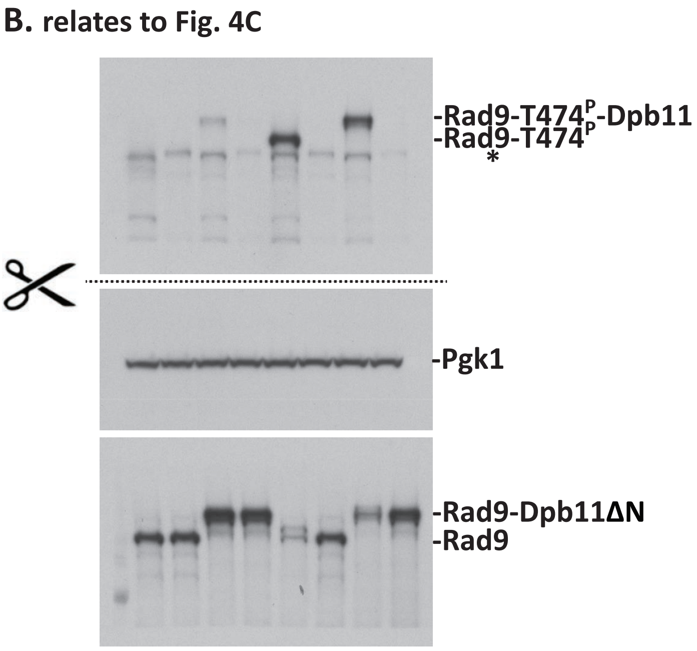

B. relates to Fig. 4C
-Rad9-T474P-Dpb11
-Rad9-T474P
*
-Pgk1
-Rad9-Dpb11ΔN
-Rad9

Figure S13

A. relates to Fig. S4D

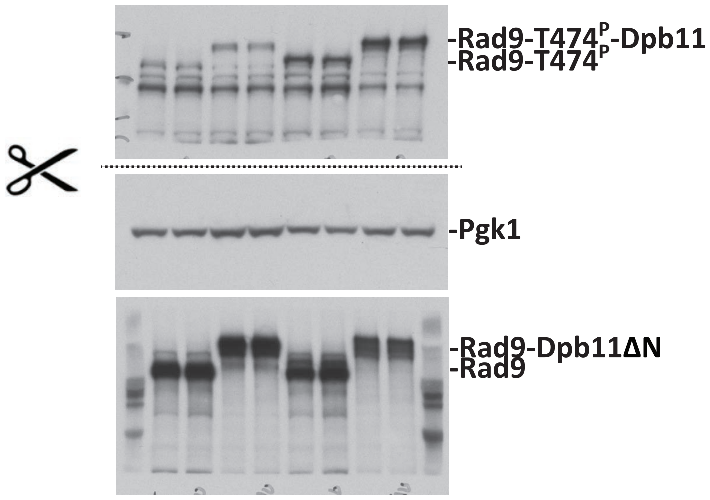

Figure S14

A. relates to Fig. 5C

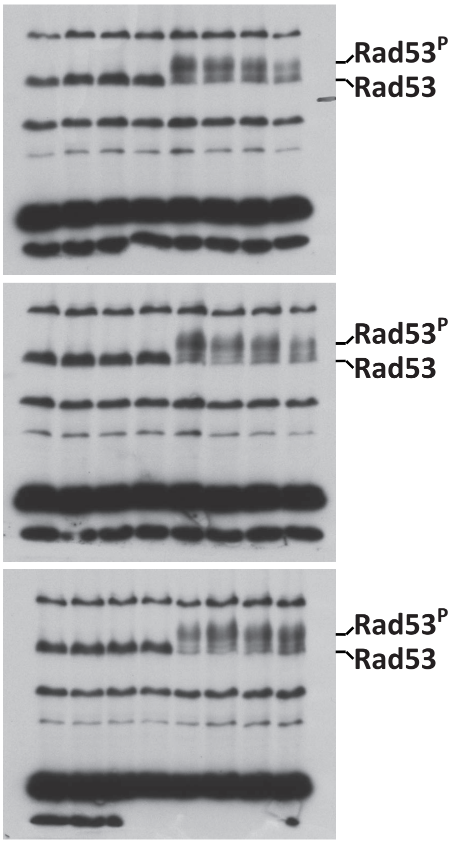

Figure S1:
Damage-induced interaction of Dpb11 and Rad9.
(A) Pulldown with recombinant GST-Dpb11 and extracts of asynchronous cells after MMS treatment shows damage-induced interaction of Rad9 and Dpb11.
(B) FACS-based DNA content measurement of experiment in Figure 1A.
(C) FACS-based DNA content measurement of experiment in Figure 1B.
(D) FACS-based DNA content measurement of experiment in Figure 1C.
(E) FACS-based DNA content measurement of experiment in Figure 1D.
(F) FACS-based DNA content measurement of experiment in Figure 1E.

Figure S2:
Involvement of checkpoint kinases in Rad9-T474 phosphorylation.
(A) FACS-based DNA content measurement of experiment in Figure 2A.
(B) FACS-based DNA content measurement of experiment in Figure 2B.
(C) FACS-based DNA content measurement of experiment in Figure 2C.
(D) Cell extracts of G2/M-arrested cells treated with phleomycin or mock-treated were probed with indicated antibodies. The Rad9-T474p phosphospecific antibody detects cell cycle-specific Rad9 phosphorylation in all mutant backgrounds. FACS-based DNA content measurement below.

Figure S3:
Involvement of Rad9-recruitment pathways in Rad9-T474 phosphorylation.
(A) FACS-based DNA content measurement of experiment in Figure 3A.
(B) *dot1Δ* cells retain S/TP phosphorylation of Rad9 in G2/M. Extracts from G2/M-arrested and phleomycin-treated cells of the indicated mutant background were probed with the indicated antibodies. FACS-based DNA content measurement below.
(C) A defect in the Rad9 TUDOR-domain (*rad9-Y798Q*) does not abolish Rad9 T474 phosphorylation S/TP phosphorylation in G2/M cells after DNA damage. Experiment as (B), FACS-based DNA content measurement below.
(D) *RAD9* mutant backgrounds impairing its recruitment to chromatin (*dot1Δ* and *rad9-Y798Q*) lead to defects in Rad9-T474 phosphorylation and Rad53 phosphorylation, when arrested in G1. Rad53 activation measured by detecting its phospho-shift on 10% SDS-gels using anti-rad53 antibodies. Right: FACS-based DNA content measurement.
(E) FACS-based DNA content measurement of experiment in Figure 3D.

Figure S4:
Influence of a Rad9-Dpb11 fusion on Rad9 function and phosphorylation.
(A) FACS-based DNA content measurement of experiment in Figure 4A.
(B) RPA ChIPs demonstrate inhibition of resection in the presence of the *RAD9-DPB11ΔN* fusion. RPA recruitment was measured at positions spanning 1.1 to 70 kb from an HO-induced DSB at the indicated time-points in G2/M arrested cells. Lower panel: DNA loss visualized by ChIP-DNA inputs (input DNA at each position relative to controls outside of the affected region). Right: FACS-based DNA content measurement.
(C) FACS-based DNA content measurement of experiment in Figure 4B.

(D) S/TP phosphorylation of Rad9 occurs in G2/M arrested cells independently of *RAD9-DPB11ΔN*. Extracts were probed with indicated phosphospecific antibodies. FACS-based DNA content measurement below.
(E) FACS-based DNA content measurement of experiment in Figure 4C.

Figure S5:
Phenotypic analysis of the *rad9-AA* mutant, deficient in binding to Dpb11.
(A) FACS-based DNA content measurement of experiment in Figure 5A.
(B) FACS-based DNA content measurement of experiment in Figure 5B.
(C) FACS-based DNA content measurement of experiment in Figure 5C.
(D) Dpb11 binding to DSBs in G1 as visualized by Dpb11-3FLAG ChIPs is abolished in *ddc1-T602* cells, but not in the *rad9-AA* cells. Dpb11 enrichment and spreading was measured starting from 1.1kb until 75kb away from an HO-induced DSB at the indicated time-points. FACS-based DNA content measurement below.

Figure S6:
(A) Original Western blots relating to Figure 1A
(B) Original Western blots relating to Figure 1B
(C) Original Western blots relating to Figure 1C
(D) Original Western blots relating to Figure 1D
(E) Original Western blots relating to Figure 1E

Figure S7:
(A) Original Western blots relating to Figure S1A

Figure S8:
(A) Original Western blots relating to Figure 2A
(B) Original Western blots relating to Figure 2B
(C) Original Western blots relating to Figure 2C

Figure S9:
(A) Original Western blots relating to Figure S2D

Figure S10:
(A) Original Western blots relating to Figure 3B
(B) Original Western blots relating to Figure 3C
(C) Original Western blots relating to Figure 3D

Figure S11:
(A) Original Western blots relating to Figure S3B
(B) Original Western blots relating to Figure S3C
(C) Original Western blots relating to Figure S3D

Figure S12:
(A) Original Western blots relating to Figure 4B
(B) Original Western blots relating to Figure 4C

Figure S13:
(A) Original Western blots relating to Figure S4D

Figure S14:
(A) Original Western blots relating to Figure 5C

Table 1:

strain	Relevant genotype	reference
W303	MATa leu2-3,112 trp1-1 can1-100 ura3-1 ade2-1 his3-11,15 [phi+]	Thomas and Rothstein, 1989
JPY923	MATa FLAG-rad53::LEU2 bar1 Δ::hisG cdc13-1 cdc15-2	Usui et al., 2008
JPY993	MATa FLAG-rad53::LEU2 bar1Δ::hisG cdc13-1 cdc15-2 rad9S1129A::URA3	Usui et al., 2008
JPY3344	MATa FLAG-rad53::LEU2 bar1Δ::hisG cdc13-1 cdc15-2 rad9-6AQ	Usui et al., 2008
YBP61	MATa RAD9-9myc::hphNT1	Pfander & Diffley, 2011
YBP109	MATa dot1Δ::kanMx4	Pfander & Diffley, 2011
YBP269	MATa ddc1-T602A::his3Mx6	Pfander & Diffley, 2011
YBP270	MATa ddc1-T602A::his3Mx6 dot1Δ::kanMx4	Pfander & Diffley, 2011
YBP366	MATa rad9Δ::natNT2 TRP1::RAD9-3FLAG::HISMx6 pep4::hphNT1	Pfander & Diffley, 2011
YBP388	MATa pep4Δ::LEU2	Pfander & Diffley, 2011
YBP390	MATa bar1Δ::TRP1	Pfander & Diffley, 2011
YBP403	MATa rad9Δ::natNT2 TRP1::rad9-3FLAG::HIS3Mx6 pep4Δ::LEU2 dot1 Δ::kanMx4	Pfander & Diffley, 2011
YBP406	MATa rad9Δ::NATNT2 TRP1::rad9AA-3FLAG::HIS3Mx6 pep4Δ::LEU2	Pfander & Diffley, 2011
YGD030	MATa rad9Δ::NATNT2 bar1Δ::HISMX6 trp1::RAD9-DPB11ΔN::TRP1	This study
YGD031	MATa RAD9-3FLAG::hphNT1 hml::pRS hmr::pRS bar1Δ::TRP1 pGal-HO::ADE3	This study
YGD032	rad9Δ::hphNT1 hml::pRS hmr::pRS bar1 Δ::TRP1 pGal-HO::ADE3	This study
YGD034	MATa rad9Δ::hphNT1 LEU2::RAD9AA-3FLAG hml::pRS hmr::pRS bar1::TRP1 pGal-HO::ADE3	This study
YGD035	MATa RAD9-3FLAG::hph dot1 Δ::kanMX4 hml::pRS hmr::pRS bar1Δ::TRP1 pGal-HO::ADE3	This study
YGD036	MATa rad9Δ::NATNT2 trp1-1::RAD9-DPB11ΔN::TRP1 mec1Δ::LEU2 tel1Δ::hphNT1 bar1Δ::HISMX6 sml1Δ::URA3	This study
YGD037	MATa trp1-1::RAD9-DPB11::TRP1 mec1Δ::LEU2 bar1Δ::HISMX6 rad9Δ::NATNT2 sml1Δ::URA3	This study
YGD038	MATa mec1Δ::LEU2 tel1Δ::NATNT2 bar1Δ::TRP1 sml1Δ::URA3	This study
YGD039	MATa rad53Δ::kanMX4 chk1Δ::NATNT2 bar1Δ::TRP1	This study
YGD040	MATa yku70::NAT rad9Δ::hphNT1 hml::pRS hmr::pRS bar1Δ::TRP1 pGal-HO::ADE3	This study

YGD041	MATa yku70::NATNT2 dot1Δ::kanMX4 hml::pRS hmr::pRS bar1Δ::TRP1 pGal-HO::ADE3	This study
YGD042	MATa RAD9-DPB11ΔN-3FLAG::hphNT1 hml::pRS hmr::pRS bar1Δ::TRP1 pGal-HO::ADE3	This study
YGD043	MATa RAD9-DPB11ΔN-3FLAG::hphNT1 dot1Δ::kanMX4 hml::pRS hmr::pRS bar1Δ::TRP1 pGal-HO::ADE3	This study
YGD044	MATa rad9Δ::hphNT1 leu2-3::Rad9AA-3FLAG::LEU2 yku70Δ::NATNT2 hml::pRS hmr::pRS bar1Δ::TRP1 pGal-HO::ADE3	This study
YGD045	MATa hml::pRS hmr::pRS bar1Δ::TRP1 pGal-HO::ade3 dpb11-3FLAG::kanMX4 rad9-AA::NATNT2	This study
YGD046	MATa hml::pRS hmr::pRS bar1Δ::TRP1 pGal-HO::ADE3 ddc1-T602A::hphNT1 DPB11-3FLAG::kanMX4 rad9-AA::NATNT2	This study
YKR112	MATa cdc28-F88G	Reusswig et al., 2016
YSB95	MATa mec1Δ::LEU2 bar1Δ::TRP1 sml1Δ::URA3	This study
YSB96	MATa rad53Δ::hphNT1 bar1Δ::TRP1 sml1Δ::URA3	This study
YSB97	MATa tel1Δ::NATNT2 bar1Δ::TRP1	This study
YSB98	MATa chk1Δ::NATNT2 bar1Δ::TRP1	This study
YSB189	MATa rad9Δ::NATNT2 pep4Δ::kanMX4 leu2-3::rad9-Y798Q-3FLAG::LEU2	This study
YSB517	MATa hml::pRS hmr::pRS bar1Δ::TRP1 pGal-HO::ADE3	Bantele et al. 2017
YSB812	MATa hml::pRS hmr::pRS bar1Δ::TRP1 pGal-HO::ADE3 dpb11-3FLAG::kanMX4	This study
YSB816	MATa hml::pRS hmr::pRS bar1Δ::TRP1 pGal-HO::ADE3 ddc1-T602A::hphNT1	This study

Table 2:

Antibody	Antigen	Source
Mouse anti-Pgk1	Pgk1	Invitrogen
Rabbit anti-Rad9	Rad9	F. Lowndes EMBO J. 1998
Rabbit anti-Rad9-S462P	Rad9 S462P peptide	Pfander, B. & J. Diffley EMBO J. 2010
Rabbit anti-Rad9-T474P	Rad9 T474P peptide	Pfander, B. & J. Diffley EMBO J. 2010
Rabbit anti-Rad53	Rad53	Abcam
Rabbit anti-RPA	Rfa1, Rfa2, Rfa3	Agrisera
Rabbit anti-FLAG	Synthetic FLAG sequence containing peptide DYKDDDDK-GC	Sigma
Mouse anti-myc	myc aa410-420	Millipore
Rabbit anti-GST-Dpb11	GST-Dpb11 555-C	Pfander, B. & J. Diffley EMBO J. 2010
